# Genomic Adaption and Mutational Patterns in a HaCaT Subline Resistant to Alkylating Agents and Ionizing Radiation

**DOI:** 10.3390/ijms22031146

**Published:** 2021-01-24

**Authors:** Reinhard Ullmann, Benjamin Valentin Becker, Simone Rothmiller, Annette Schmidt, Horst Thiermann, Hanns Leonhard Kaatsch, Gerrit Schrock, Jessica Müller, Julia Jakobi, Richard Obermair, Matthias Port, Harry Scherthan

**Affiliations:** 1 Bundeswehr Institute of Radiobiology Affiliated to the University of Ulm, Neuherbergstr. 11, D-80937 Munich, Germany; HannsLeonhardKaatsch@bundeswehr.org (H.L.K.); gerrit2686@hotmail.com (G.S.); Jessica4Mueller@bundeswehr.org (J.M.); JuliaJakobi@bundeswehr.org (J.J.); richard.obermair@web.de (R.O.); MatthiasPort@bundeswehr.org (M.P.); harryscherthan@bundeswehr.org (H.S.); 2 Bundeswehr Central Hospital, Department of Radiology and Neuroradiology, Rübenacherstrasse 170, D-56072 Koblenz, Germany; benjamin3becker@bundeswehr.org; 3 Bundeswehr Institute of Pharmacology and Toxicology, Neuherbergstr. 11, D-80937 Munich, Germany; Simone1Rothmiller@bundeswehr.org (S.R.); annette.schmidt@unibw.de (A.S.); HorstThiermann@bundeswehr.org (H.T.)

**Keywords:** chronic exposure, DNA alkylation, genome rearrangements, HaCaT, irradiation, mutational signatures, oxidative stress, resistance, sulfur mustard

## Abstract

Sulfur mustard (SM) is a chemical warfare agent that can damage DNA via alkylation and oxidative stress. Because of its genotoxicity, SM is cancerogenic and the progenitor of many chemotherapeutics. Previously, we developed an SM-resistant cell line via chronic exposure of the popular keratinocyte cell line HaCaT to increasing doses of SM over a period of 40 months. In this study, we compared the genomic landscape of the SM-resistant cell line HaCaT/SM to its sensitive parental line HaCaT in order to gain insights into genetic changes associated with continuous alkylation and oxidative stress. We established chromosome numbers by cytogenetics, analyzed DNA copy number changes by means of array Comparative Genomic Hybridization (array CGH), employed the genome-wide chromosome conformation capture technique Hi-C to detect chromosomal translocations, and derived mutational signatures by whole-genome sequencing. We observed that chronic SM exposure eliminated the initially prevailing hypotetraploid cell population in favor of a hyperdiploid one, which contrasts with previous observations that link polyploidization to increased tolerance and adaptability toward genotoxic stress. Furthermore, we observed an accumulation of chromosomal translocations, frequently flanked by DNA copy number changes, which indicates a high rate of DNA double-strand breaks and their misrepair. HaCaT/SM-specific single-nucleotide variants showed enrichment of C > A and T > A transversions and a lower rate of deaminated cytosines in the CpG dinucleotide context. Given the frequent use of HaCaT in toxicology, this study provides a valuable data source with respect to the original genotype of HaCaT and the mutational signatures associated with chronic alkylation and oxidative stress.

## 1. Introduction

Sulfur mustard (SM) is infamous for its use as a chemical warfare agent on the battlefield of Ypres during the First World War in 1917 and in several other more recent military conflicts. Even currently, SM poses a continuing threat, as it is probably the most widely distributed chemical weapon to date [1,2,3].

A variety of mechanisms have been proposed to explain the tissue-dependent cytotoxic effects of SM [4]. With respect to its genotoxicity, the high bifunctional alkylating activity of SM can lead to DNA adducts and crosslinks, particularly involving the N7-position of deoxyguanosine and, to a lesser extent, the N3-position of deoxyadenosine [5,6,7,8,9,10,11]. In addition, DNA can be damaged by oxidative stress due to SM-induced depletion of endogenous antioxidants [12,13,14,15]. One outcome of SM-induced DNA damage can be an increase in PARP activity resulting in NAD+ depletion, decreased glycolysis, and subsequent protease release, leading to reduced cellular fitness [16].

As a consequence of its genotoxicity, SM is a well-established risk factor for bronchial carcinoma and several other tumor types [17,18,19,20,21,22]. At the same time, SM is the progenitor of many chemically and/or functionally related chemotherapeutics. Already in 1943, nitrogen mustard, a SM derivative, was used to treat a patient with non-Hodgkin lymphoma [23,24]. Since then, nitrogen mustards have been further improved in terms of efficacy and side effects and are regularly used in clinics for the treatment of various types of cancer [25]. The flipside of therapy with this kind of alkylating drug is that tumor cells can acquire resistance [26,27] and patients can develop therapy-associated secondary tumors later on [28]. These complications underscore the need to obtain more detailed knowledge about how alkylating drugs can alter the genomic landscape and in which way these changes might contribute to the development of resistance and secondary tumors.

In previous work, we generated a cell line resistant to SM (HaCaT/SM) [29,30]. The resistant cell population originates from the highly popular keratinocyte cell culture model HaCaT, which is an immortal hypotetraploid cell line that is nontumorigenic and has retained its capacity to differentiate [31,32]. HaCaT displays a rather stable karyotype [33,34] and adaptability to genotoxic stressors [35,36]. The resistant cell line HaCaT/SM emerged after chronic exposure of parental HaCaT cells to increasing doses of SM over a period of 40 months [30]. Further studies demonstrated that resistance was not limited to SM but extended to further nine cytostatics, with several of them currently used in tumor therapy [37]. This makes HaCaT/SM a suitable model to study the spectrum of genetic alterations associated with prolonged exposure and subsequent resistance to alkylating agents and oxidative stress. Using a broad spectrum of methods, ranging from classical cytogenetics to whole-genome sequencing, we performed a comprehensive analysis of chromosomal aberrations and point mutations in HaCaT and its SM-resistant derivative. We present evidence that chronic SM exposure promoted the expansion of a hyperdiploid cell population with an elevated rate of structural chromosomal aberrations and a characteristic mutational signature.

## 2. Results

### 2.1. Sulfur Mustard Exposure Promoted Clonal Expansion of a Hyperdiploid Cell Population with Higher Tolerance to Ionizing Radiation

**Figure 1:**
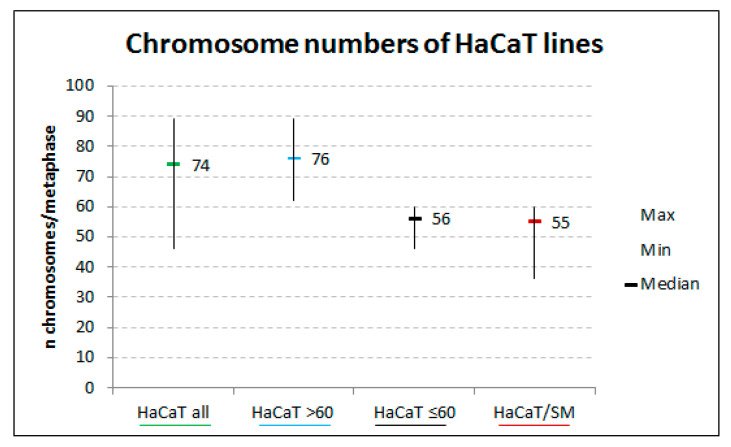
Chromosome numbers in HaCaT/sulfur mustard (SM) and HaCaT. HaCaT parents (all) and subclones (≤60 and >60 chromosomes) are shown. The bars indicate the median. The whiskers indicate the range (min, max). Numbers are based on the analysis of 75 metaphases/cell line.

Previous studies showed that SM resistance in HaCaT/SM went along with higher proliferative capability, increased cell survival, and smaller cell nuclei [29]. To scrutinize whether this reduction in nuclear size is due to either higher chromatin compaction or lower DNA content per cell, we prepared chromosome spreads of the parental and the resistant cell line and determined their average chromosome number. It was found that the parental cell line HaCaT was composed of at least two cell populations, one with 76 (in 76% of cells) and one with 56 chromosomes (in 24% of cells) on average, whilein HaCaT/SM the distribution of chromosome numbers cells suggests a more homogenous cell population with a median of 55 chromosomes (Figure 1). This indicates that chronic exposure to SM induced positive selection of the hyperdiploid cell population at the cost of the hypotetraploid one that dominated the parental cell line. This change in ploidy was in line with DNA content measurements of the two cell lines (Supplementary Table S1).

**Figure 2:**
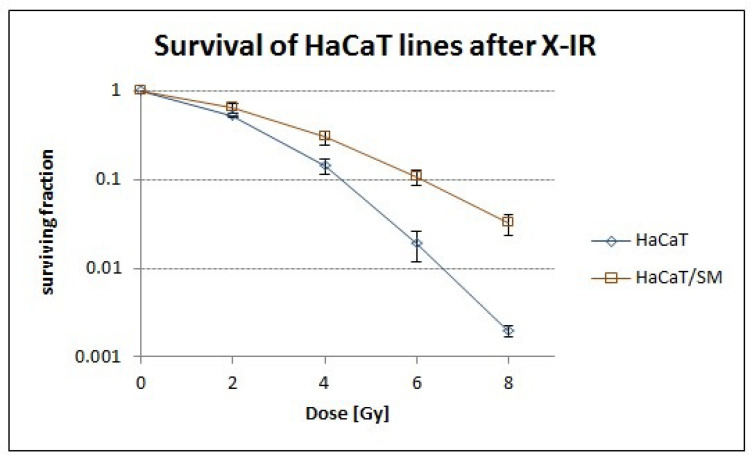
Survival of HaCaT after irradiation. Cellular survival after increasing doses of X-ray ionizing radiation (IR) shows a radioresistant phenotype of the HaCaT/SM cell line, especially at higher doses, as revealed by clonogenic assay (data are based on three replicate experiments ± SD).

Previous studies revealed that the mechanisms mediating resistance in HaCaT/SM also protect from other chemical stressors [37]. Through exposure to increasing doses of X-rays, we were also able to demonstrate significant differences in cellular survival in response to ionizing radiation (Figure 2). Exposure to both ionizing radiation (IR) and alkylating agents can result in increased levels of reactive oxygen species (ROS), suggesting a central role of oxidative stress response in the cross-resistance of HaCaT/SM.

### 2.2. Shared and Unique Structural Chromosomal Aberrations in HaCaT and HaCaT/SM

**Figure 3:**
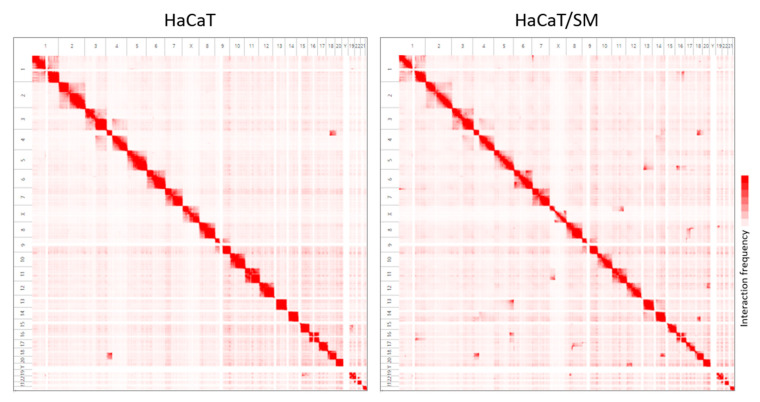
Genome-wide detection of chromosomal translocations by Hi-C interaction matrices for HaCaT (**left**) and HaCaT/SM (**right**). Red color saturation corresponds to the number of Hi-C interactions between chromosomal regions and, hence, their probability of spatial proximity within the nucleus. Chromosome numbers are given at the top and to the left of each matrix. See text for further explanations. Juicebox was employed for visualization of Hi-C data [43]. Enlarged views of t(4;18) and t(19;22) are exemplarily provided in Supplementary Figure S1. Genomic coordinates of translocations are given in Supplementary Table S2.

Next, we performed a genome-wide screen for chromosomal translocations. Translocations drastically change the spatial proximity of chromosomes within the nucleus. This fact can be used to infer chromosomal translocation partners and chromosomal breakpoints on the basis of analysis of chromosome interaction probabilities by means of Hi-C [38,39,40,41,42] (Figure 3; Supplementary Table S2).

**Figure 4:**
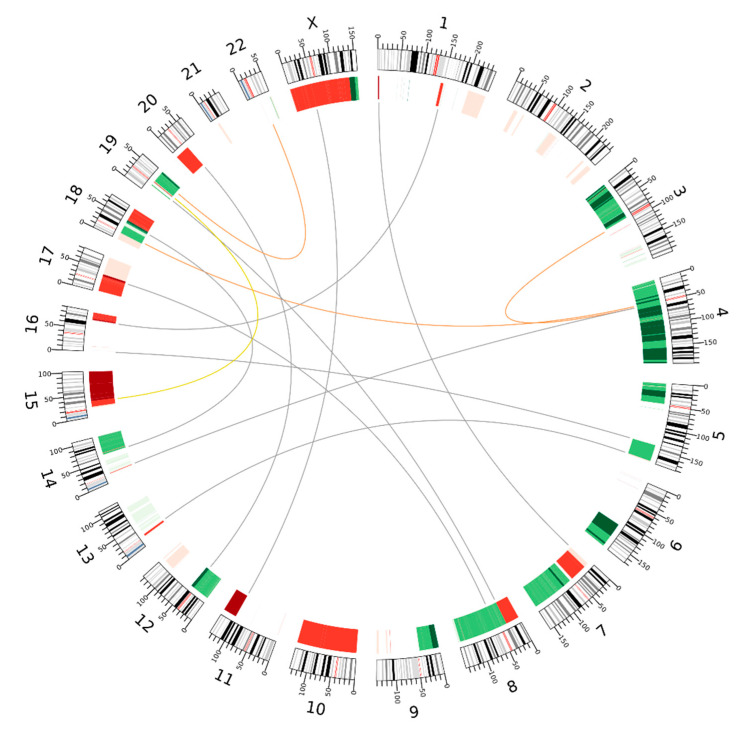
Circos plot of chromosomal translocations and DNA copy number differences in HaCaT and HaCaT/SM. Copy number differences between HaCaT/SM and HaCaT are plotted next to the radially oriented chromosome ideograms. Green indicates higher and red indicates lower DNA copy number in HaCaT/SM relative to HaCaT. The degree of copy number change as defined by their array CGH log_2_ ratio is indicated by three different grades of color saturation to enable the distinction of moderate from high copy number changes (dark color: log_2_ threshold ±0.5, medium color: log_2_ threshold 0.2, and light color: log_2_ threshold 0.12; see Section 4 for details). Orange links within the ideogram show translocations common to both cell lines, whereas yellow and gray links show those specific to HaCaT and HaCaT/SM, respectively. Coordinates of translocation breakpoints are provided in Supplementary Table S2. Visualization was done by means of Circos [46].

In addition to the three translocations present in both cell populations, Hi-C interaction matrices revealed seven exclusive translocations in HaCaT/SM and one translocation t(15;19) specific to HaCaT. The latter was most likely originally present in both cell lines before it was eliminated by chromosomal loss in HaCaT/SM (Figure 4). Two of the three translocations shared, t(3;4) and t(4;18), were previously reported by Boukamp and colleagues as structural abnormalities already detectable at passage five of HaCaT [31,33]. In contrast, the seven HaCaT/SM-specific translocations do not match any translocation reported by these authors, whether for early or later passages of HaCaT.

We next analyzed chromosomal gains and losses in HaCaT and HaCaT/SM by array Comparative Genomic Hybridization (array CGH) and quantitative analysis of whole-genome sequencing reads. The copy number alterations identified using both methods largely overlapped, although we also detected copy number alterations exclusively present in one of the two datasets. These differences are most likely due to the fact that the DNA used for array CGH analysis was isolated from a later passage than the DNA used for whole-genome sequencing. All DNA copy segments defined by circular binary segmentation [44] of array CGH-derived log_2_ ratios are provided in Supplementary Table S3.

Both HaCaT and HaCaT/SM karyotypes were characterized by complex patterns of chromosomal changes. Comparative analysis revealed extensive karyotypic differences between the two HaCaT cell populations ranging from gross chromosomal rearrangements encompassing several megabases to submicroscopic changes, partly overlapping fragile sites such as Fra1B, Fra2K, Fra3B, and Fra16D. Noteworthy, the gene *WWOX* mapping to Fra16D harbored two deletions. The first one, chr16: 78,371,638–78,384,899 (hg19), was also present in a fraction of parental HaCaT cells, while the second one, chr16: 78,465,122–78,622,373 (hg19), was exclusively found in HaCaT/SM. We also observed elimination of the surplus chromosome 15 in HaCaT/SM, which was present in the parental cells. A numerical loss of chromosome 15 was previously reported in the context of malignant transformation of HaCaT by HRAS [34,45].

**Figure 5:**
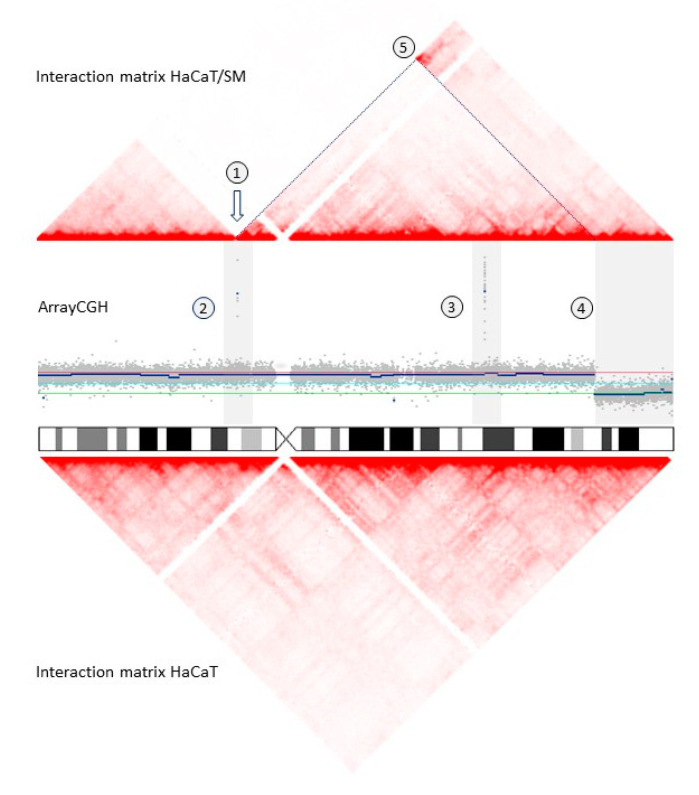
Changes in DNA copy number and chromatin conformation suggest complex rearrangement of chromosome X. Triangular Hi-C interaction matrices for HaCaT/SM and HaCaT are plotted above and below the array CGH log_2_ ratio plots. Increased red color saturation at the tip of a triangle connecting two genomic positions at the base line indicates higher interaction probability (exemplarily highlighted in the Hi-C results of HaCaT/SM by dashed lines): (1) translocation breakpoint t(11;X); (2) small deletion (chrX: 48,928,825–48,942,873) adjacent to the translocation breakpoint; (3) deletion chrX:109,358,110–109,515,685; (4) duplication chrX:148,572,165–155,257,126; (5) increased chromosomal interaction probability indicating spatial proximity of the chromosomal duplication and translocation breakpoints (highlighted by gray dashed lines). Chromosome coordinates refer to hg19.

A great proportion of HaCaT/SM specific copy number alterations were adjacent to translocation breakpoints (Figure 4). On the example of chromosome X, integrative analysis of Hi-C and array CGH data revealed that seemingly simple and independent structural aberrations were connected and resulted in considerable conformational changes in the affected chromosome (Figure 5). This rearrangement of chromosome X was exclusively present in array CGH and Hi-C data, but absent in whole-genome sequencing (WGS) data, indicating that this change occurred at a later stage of SM exposure.

### 2.3. Genomic Characteristics at Sites of DNA Double-Strand Breaks

According to the quantitative analysis of whole-genome sequencing data, we created a manually curated dataset of 75 HaCaT/SM-specific chromosomal breakpoints resolved at a single-base level (Supplementary Table S4). As for non-B-conformations, seven of those breakpoints mapped to inverted repeats and two were located within direct repeats. No G-quadruplex motifs were identified at or near the breakpoints (100 bp up- and downstream interval). We submitted 100 bp sequence intervals surrounding the breakpoints to MEME Suite [47] for sequence motif discovery. Three sequence motifs were significantly overrepresented (Supplementary Figure S2). Yet, none of the motifs had a specific location with respect to the chromosomal breakpoint, and two of the three significantly overrepresented motifs discovered by MEME Suite were identified in sequence intervals that mapped to either SINEs or LINEs. In order to check whether the overlap with repetitive sequences exceeds what can be expected by chance, we employed the R package GenometriCorr [48]. Although 33 breakpoints intersected repetitive elements, neither overlap probability nor relative distance to repetitive elements differed significantly from expectation (Kolmogorov–Smirnov test of normal distribution of relative distances *p* = 0.763; projection test *p* = 0.464).

### 2.4. Genome-Wide Distribution of Single-Nucleotide Variants

A comparison of single-nucleotide variants (SNV) called from the WGS datasets of HaCaT and HaCaT/SM revealed that 3,974,694 SNVs were common to both cell lines, while 610,408 and 199,342 SNVs were exclusively found in the progenitor and the resistant subline, respectively (read statistics of WGS data comprising the numbers of uniquely mapped reads, mean read length, coverage, and sequencing depth are summarized in Supplementary Table S5). As expected, and exemplarily depicted for chromosome 8 (Supplementary Figure S3), many of these presumptive unique SNVs showed strong correlation with DNA copy number state. Therefore, search for HaCaT/SM-specific mutational patterns was limited to a subset of SNVs as detailed in Section 4 to minimize the misleading influence of cell line-specific chromosomal gains and losses on the definition of HaCaT/SM-specific mutational signatures.

**Figure 6:**
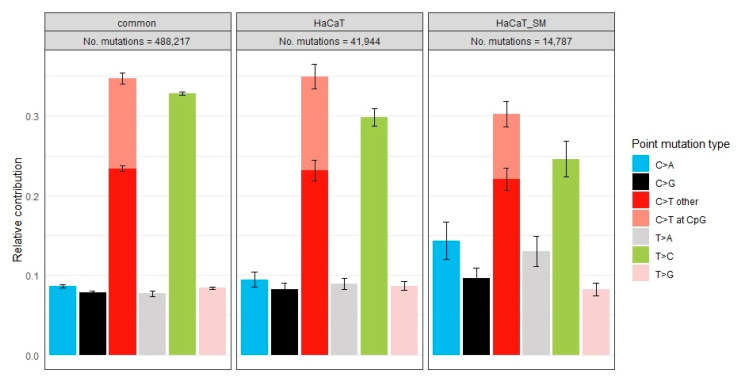
Mutation type frequency plot of common and unique single-nucleotide variants (SNVs). Numbers of mutations are given at the top. The bars represent the relative contribution of each mutation type with reference to the pyrimidine of each basepair. A color legend is provided to the right. The error bars indicate the 95% confidence interval.

Altogether, this subset of SNVs, mapping to eight chromosomal regions lacking prominent differences in DNA copy number and local bias in mutation frequency between the two cell populations, comprised 488,217 common, 41,944 HaCaT-specific, and 14,787 HaCaT/SM-specific SNVs. As can be inferred from the mutation type frequency plot provided in Figure 6, with reference to the pyrimidine of the DNA double strand, the set of HaCaT/SM-specific SNVs was characterized by a higher rate of C > A and T > A transversions at the cost of T > C and C > T transitions, the latter particularly in the CpG dinucleotide context. An increase in C > A transversions can also be caused by 8-oxoguanine modifications arising in the course of sequencing library preparation [49]. As this artefact should affect both DNA strands with similar frequency, we tested the strand-specificity of all genic SNVs. This analysis revealed a significant bias of C > A transversions toward the transcribed strand (Supplementary Figure S4), making a preparation-derived artefact unlikely.

### 2.5. Mutational Signatures

**Figure 7:**
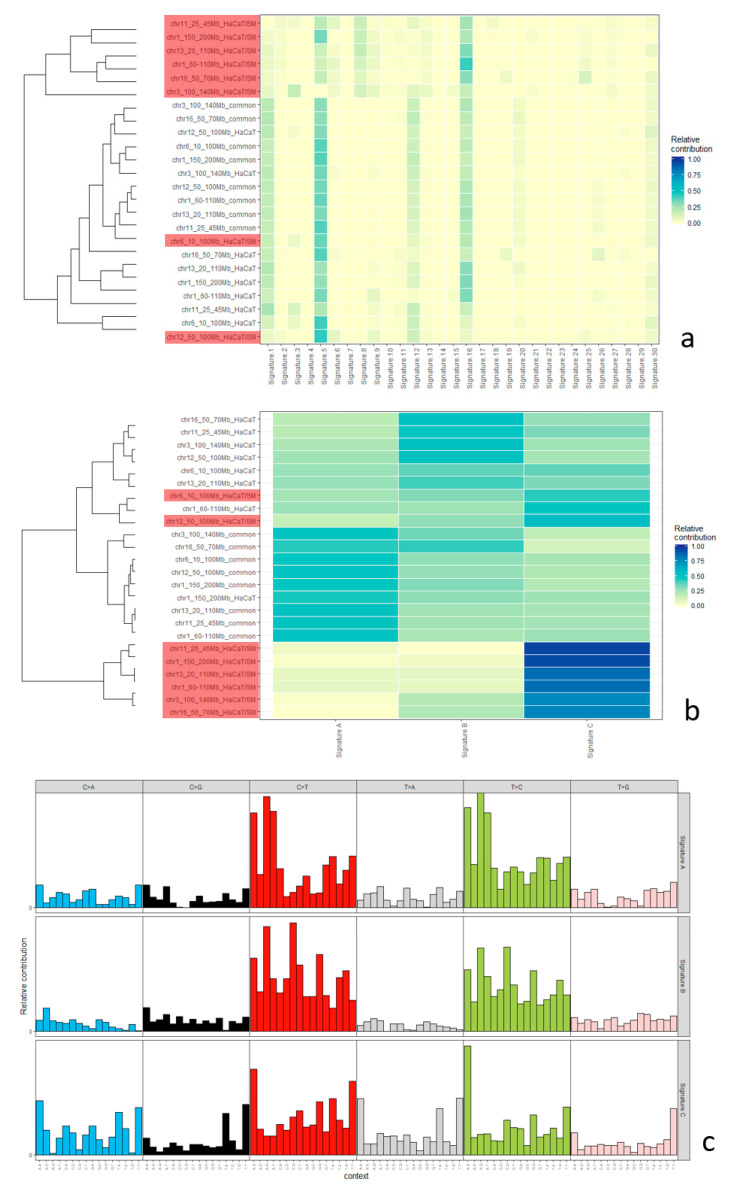
Mutational signatures identified in HaCaT/SM-specific SNV (next page). (**a**) Unsupervised hierarchical clustering based on the relative contribution of 30 previously published mutational signatures. The numbering of signatures refers to Mutation Signatures v2—COSMIC. Names given to the left of each heatmap indicate the chromosomal region of the respective SNV set and whether the SNV set is common or unique to HaCaT and HaCaT/SM, respectively. HaCaT/SM specific SNV sets are additionally highlighted in red. (**b**) Unsupervised hierarchical clustering based on the relative contribution of mutational signatures as extracted by non-negative matrix factorization from the 24 SNV sets representing common and unique SNVs in HaCaT and HaCaT/SM, respectively. HaCaT/SM-specific SNV sets are highlighted in red. (**c**) Relative contribution of each mutation type with reference to the pyrimidine of each basepair for the three signatures shown in (**b**). Each mutation type is shown in the trinucleotide context, i.e., in the middle of a trinucleotide with all possible combinations of neighbors to the left and right. All images were generated by means of the *Mutational Patterns* software package [54].

The mutational landscape of a given cell is shaped by the additive contribution of various mutational processes. Each of these processes can exhibit bias with regard to mutation type and sequence context. The term “mutational signatures” has been coined for these distinct mutational footprints. In most tumors, the overall mutational landscape is a composite of several mutational signatures [50,51]. At first, we tested whether HaCaT/SM-specific SNV subsets derived from these eight selected chromosomal regions described above can be distinguished from common and HaCaT-specific SNV subsets. Unsupervised hierarchical clustering of the 24 SNV subsets (common, HaCaT, and HaCaT/SM for eight chromosomal regions) based on the relative contribution of previously published 30 mutational signatures (Mutation Signatures v2–Cosmic) [52] grouped six out of eight HaCaT/SM SNV subsets in one cluster (Figure 7a). In line with the lower prevalence of C > T transitions at CpG dinucleotides in HaCaT/SM, age-associated signature #1 contributed less to HaCaT/SM-specific SNVs when compared to its contribution to common and HaCaT specific SNVs. Although HaCaT is a keratinocyte cell line, we failed to identify a major contribution of ultraviolet (UV) exposure-associated signature # 7 (Figure 7a).

In a next step, we tried to define a mutational signature most representative for the set of HaCaT/SM-specific SNVs. For this purpose, the non-negative matrix factorization (NMF) algorithm [53] implemented in the software package *Mutational Patterns* [54] was employed to extract three mutational signatures from the 24 SNV subsets. The extraction of three mutational signatures should account for the fact that HaCaT/SM-specific SNVs are unlikely induced by SM alone, but include at least a fraction of mutations caused by cell culture conditions and the continuous action of mutational processes already present in HaCaT. However, it is clear that three mutational signatures cannot cover the full complexity of mutational events and, therefore, should be seen as a compromise helping to avoid overstretching the analysis by too many signatures.

Unsupervised hierarchical clustering based on the relative contribution of these three signatures to the overall mutational spectrum of both HaCaT populations clustered the same six out of eight selected chromosomal regions as observed in the clustering experiment described above (Figure 7b). The mutational signature characteristic for HaCaT/SM was dominated by an increased rate of transversions, thereby reflecting the mutation type bias already identified in the HaCaT/SM-specific SNV set (Figure 7c).

## 3. Discussion

In this study we undertook a genome-wide characterization of chromosomal rearrangements and mutational patterns in the SM-resistant HaCaT cell line. We identified genetic changes associated with chronic exposure to SM, which may impact resistance toward various alkylating agents and ionizing irradiation.

### 3.1. Change in Ploidy and Structural Chromosomal Aberrations

We observed that chronic exposure of the parental HaCaT cell line to SM induced the expansion of a hyperdiploid subclone at the cost of the originally prevailing hypotetraploid cell population. This reversed the initial polyploidization at passage 5 of the HaCaT cell line reported by Boukamp and colleagues, which was deemed essential for autonomous growth of HaCaT [31,55], but apparently no longer for the hyperdiploid HaCaT/SM line. The expansion of a cell clone with lower DNA content was rather unexpected in light of previous reports that linked polyploidization to increased tolerance and adaptability toward genotoxic stressors [56].

In addition to differences in chromosome number, both cell lines also considerably differed with respect to structural chromosomal aberrations. The rather high number of chromosomal translocations exclusive to HaCaT/SM tempted us to speculate that these aberrations might not have evolved gradually and independently, but in a saltatory and interdependent manner, as described for a specific aberrational pattern termed chromoplexy [57]. Yet, we failed to identify any complex chained translocations, the main characteristic of chromoplexy. Notably, the majority of translocation breakpoints were associated with changes in DNA copy number. Unless these unbalanced translocations are due to a nonreciprocal mechanism of interchromosomal DNA double-strand break repair, this observation suggests that a considerable fraction of translocations preceded the numerical changes in chromosomes, which then under selection pressure wiped out one of the derivative chromosomes.

**Figure 8:**
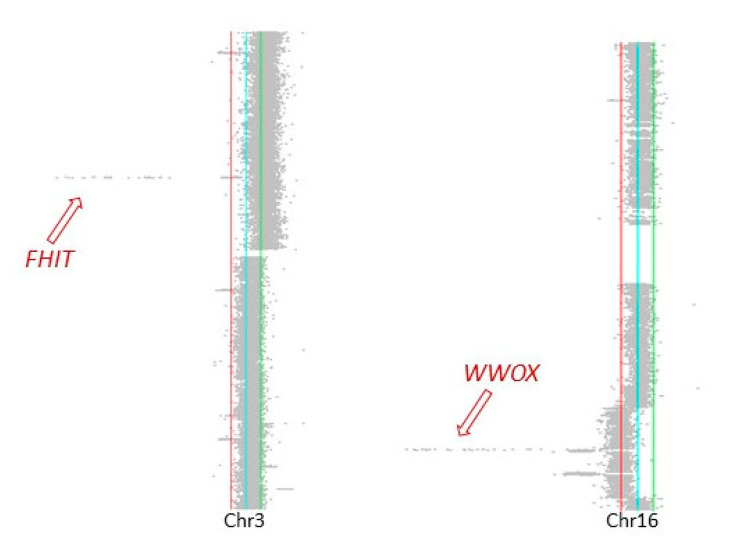
Focal deletions of *FHIT* and *WWOX* due to expression of FRA3B and FRA16D. Array CGH results for HaCaT versus HaCaT/SM for chromosome 3 and 16. Green and red lines indicate log_2_ ratios of 0.4 and −0.4, respectively. Arrows point to the deletions encompassing *FHIT* and *WWOX*.

It remains unsure to what extent these genetic changes can be directly attributed to SM exposure or alternatively represent pre-existing mutations that became detectable by SM-induced clonal selection. Either way, the high number of structural chromosomal aberrations points toward a compromised or altered DNA double-strand break repair in HaCaT/SM. Spot-checking at the level of focal chromosomal aberrations unearthed focal copy number alterations affecting several genes implicated in DNA damage response and repair, including *FHIT*, *RAD18*, *RAD51b*, and *WWOX*. Of note, the two genes lost due to the expression of two common fragile sites (Figure 8), *FHIT* (FRA3B) and *WWOX* (FRA16D), have already been discussed in the context of DNA repair pathway choice, altered efficiency of homology-dependent DNA repair, and replication-stress-induced genomic instability, respectively, as well as resistance to irradiation and various chemical stressors [58,59,60].

### 3.2. Distribution of Single-Nucleotide Variants and Mutational Signatures

The number of SNVs exclusively identified in HaCaT/SM was ~3 times lower compared to those specific to HaCaT. This rather unexpected low rate of exclusive SNVs in the HaCaT/SM genome might be a consequence of reduced cellular heterogeneity as a result of SM-induced clonal selection and/or the lower cellular DNA content in the hyperdiploid cell population. Mutation type frequencies and comparison to a collection of 30 mutational signatures (Mutation Signatures v2—Cosmic) indicate that the mutational mechanism involving deamination of 5-methylcytosines, a signature typically associated with age, contributed less to the set of HaCaT/SM-specific SNVs when compared to SNVs common to both cell lines or exclusively detected in HaCaT. Surprisingly for keratinocyte-derived cell lines and despite the presence of UV-typical mutations in *TP53* [61], we failed to prove any prominent impact of UV-associated Cosmic Signature #7 both in HaCaT and in HaCaT/SM, which might be due to the fact that HaCaT cells originate from a site not extensively exposed to sunlight [31].

HaCaT/SM-specific SNVs were further characterized by an increased rate of transversions. In particular, C > A transversions have recently been linked to the generation of 8-oxo-guanine in the course of sequencing library preparation [49]. However, a major influence of this technical artefact on the differences in C > A mutation frequency observed here is rather unlikely, given the significant strand bias of C > A changes and the fact that both cell lines were analyzed using the very same protocols and bioinformatics pipeline. In vivo, C > A transversions are frequently caused by oxidative stress, either by mispairing or by compromised base excision repair of 8-oxo-guanine or formamidopyrimidine-guanine (Fapy-G), an alternative oxidation product of guanine [62,63]. In accordance, Kucab and colleagues observed a dominance of C > A transversions in various trinucleotide contexts after triggering oxidative stress in stem cells by means of potassium bromate. Interestingly, the same authors hardly observed C > A transversions when exposing the same stem cells to various alkylating agents [64]. This may suggest that not alkylation but oxidative stress is the main driver of point mutations associated with SM exposure. Noteworthy in this context is our observation that HaCaT/SM is less sensitive to irradiation, which supports the idea that resistance of HaCaT/SM is mainly based on a better way to handle oxidative damage that is also a feature of ionizing radiation exposure. A considerable contribution of oxidative stress to the mutational burden would also be of relevance in the context of chemotherapy with alkylating agents, as these treatments have already been reported to induce oxidative stress [65].

Yet, there are several uncertainties attached to speculations on the main source of point mutations in HaCaT/SM. Bulky DNA adducts can also lead to depurinization and consequently transversions (reviewed in [66]), and in vitro experiments have demonstrated that N7 alkylation of guanine can also result in Fapy-G lesions [62,67,68,69]. Moreover, the mutational pattern generated by chronic exposure of HaCaT/SM, with extensive chance of adaption and selection, might differ from that induced by acute intoxication of nonadapted cells. It is also likely that a fraction of the observed mutations emerged independently of SM exposure with several of them already being present in some cells before SM administration. In any case, the high frequency of both C > A and T > A transversion is suggestive of an increased occurrence of apurinic sites, as a consequence of either oxidative damage repair or alkylation-induced DNA repair, which are then preferentially complemented with adenine following the so-called A-rule [70,71,72,73].

In summary, we described the complex genomic changes associated with chronic exposure of the keratinocyte cell line HaCaT to sulfur mustard. In addition to a better understanding of the genetic consequences of prolonged treatment of cells with an alkylating and oxidative stress provoking agents, our findings remind of the fact that toxicological long-term experiments can drastically alter the genetic characteristics of this highly popular cell culture test system and most likely many others.

## 4. Materials and Methods

### 4.1. Cultivation and Authentication of HaCaT

HaCaT cells were kindly provided by Prof. Dr. N. Fusenig (German Cancer Research Center, Heidelberg, Germany) and cultivated in Dulbecco’s modified Eagle medium supplemented with glutamine and 10% fetal bovine serum (Gibco, Carlsbad, CA, USA). Cells were maintained at 37 °C and 5% CO_2_ in a humidified atmosphere. For cell passaging, HaCaT cells were detached with 0.05% trypsin in 1 mM EDTA (Gibco, Carlsbad, CA, USA) for 5 min and reseeded in fresh culture medium twice a week. The generation of HaCaT/SM is described in Schmidt et al. [30].

Authentication of HaCaT and its SM-resistant subline was based on the reidentification of cytogenetic aberrations as reported in the original description of HaCaT [31] and HaCaT-specific *TP53* mutations identified by Lehmann and colleagues [61]. The common origin of HaCaT and HaCaT/SM was further confirmed by evaluating the extent of shared single-nucleotide variants (SNVs) and DNA copy number changes. PCR testing revealed that both cell lines were infected with mycoplasma during an advanced stage of the experiments, which is, however, not considered to have a major influence on the results since the contamination affected both cell lines used for the comparisons.

### 4.2. Chromosome Preparation and Quantification of DNA Content

The HaCaT parental cell line and the SM-resistant cell line HaCaT/SM [30] in exponential growth were arrested at metaphase with 0.05 mg/mL Colcemid (Gibco, Carlsbad, CA, USA) for 2 h and then harvested by treatment with trypsin. Chromosome spreads were obtained according to standard acetic acid/methanol (1/3) fixation protocols [74]. Slides were stored at 22 °C until use. DAPI-stained metaphases were analyzed for chromosome number using a Metafer4 imaging system (MetaSystems).

For quantification of DNA content, 1.5 × 10^6^ cells of HaCaT and HaCaT/SM were used, respectively. DNA isolation was performed with a Gentra Puregene Cell Kit (Qiagen, Hilden, Germany) according to the manufacturer’s protocol. Briefly, cell pellets were lysed in 300 µL of cell lysis solution for 10 min at room temperature (RT). After addition of 100 µL protein precipitation solution, incubation on ice for 5 min, and centrifugation (3 min, 16,000× *g*), the supernatant was transferred into a new tube, and 300 µL of pure isopropanol was added. After another centrifugation, the pelleted DNA was washed with 300 µL of 70% ethanol by centrifugation. The DNA pellet was air-dried and rehydrated in 100 µL of DNA hydration solution for 1 h at 65 °C. DNA concentration was determined by NanoDrop 8000 (Thermo Fisher Scientific, Waltham, MA, USA) in biological duplicates from three independent experiments. HaCaT and HaCaT/SM were compared by the unpaired two-sample Wilcoxon test and *p*-values < 0.05 were considered statistically significant.

### 4.3. Whole-Genome Sequencing and Variant Calling

Whole-genome sequencing and primary data analysis comprising quality trimming, mapping, and variant calling were done by a commercial core facility (Eurofins, Ebersberg, Germany). In brief, 2 × 125 bp reads were generated for HaCaT and HaCaT/SM on a HiSeq2500 (Illumina, San Diego, CA, USA). Adapter sequences and low-quality bases (Phred < 20) were clipped off by means of Trimmomatic [75]. Reads < 40 bp after clipping were excluded from further analysis. Remaining reads were mapped to Hg38 employing BWA-MEM using the default settings [76]. Duplicate reads were removed and variants were called with VARScan 2.3 [77]. Common variants were distinguished from those exclusively present in HaCaT or HaCaT SM by means of the *vcf-isec* command implemented in VCF Tools [78].

### 4.4. Analysis of DNA Copy Number Changes

DNA copy number changes in HaCaT and HaCaT/SM were analyzed by means of array CGH and quantitative analysis of whole-genome sequencing data. For comparability of array CGH data, which referred to hg19, WGS data of HaCaT and HaCaT/SM were remapped to hg19 by means of BWA-MEM [76] and quality-filtered (>q30) using Samtools [79]. ACE [80] and its included functions of QDNASeq [81] were used for estimation of DNA copy number according to genomic binning of GC bias-corrected read counts with a bin size of 1000 bp. DNA used for array CGH was isolated from a later cell passage with longer exposure to SM than the DNA used for whole-genome sequencing. In the first two array CGH experiments, DNA isolated from the two HaCaT populations was separately hybridized against reference DNA derived from GM12878, which is a lymphoblastoid cell line that lacks gross structural chromosomal aberrations. GM12878 was obtained from the NIGMS Human Genetic Cell Repository at the Coriell Institute for Medical Research (Repository number GM12878). In a third experiment, HaCaT and HaCaT/SM were hybridized against each other in order to directly verify DNA copy number differences between these two HaCaT cell populations. Labeling of DNA and hybridization onto a 400 k SurePrint G3 Human CGH Array (Agilent; 4448 A) were performed according to the manufacturer’s recommendations. Feature Extraction Software 12.0.3.1 was employed for primary data analysis. Circular Binary Segmentation [44] was performed with DNACopy [82] implemented in GenomeCAT [83]. In order to distinguish moderate from high DNA copy number alterations in the Circos plot presented in Figure 4, we categorized chromosomal changes according to their log_2_ ratio shift (thresholds for each fragment defined by Circular Binary Segmentation: ±0.12, ±0.2, and ±0.5). IGV [84] and GenomeCAT [83] were used to visualize the results. All array CGH data refer to genome release hg19. Coordinates of fragile sites were taken from the HumCFS database [85].

### 4.5. Genomic Characteristics at Sites of DNA Double-Strand Breaks

For identification of chromosomal breakpoints at single-base resolution, binning results of WGS data (see above) were plotted in the IGV browser and screened by visual inspection for reads overlapping possible breakpoints. Only breakpoints confirmed by three independent breakpoint-spanning reads exclusively present in HaCaT/SM were considered for further analysis, which was mainly based on R packages downloaded from the Bioconductor depository (https://bioconductor.org/). BED files were transformed into GenomicRange objects using the import function of Rtracklayer [86]. DNAShapeR [87] was employed to automatically retrieve the sequences surrounding the breakpoints as FASTA files. These files were then uploaded to the webtool MEME suite for identification of significantly overrepresented sequence motifs (http://meme-suite.org) [47]. Statistical significance of breakpoint overlap with repetitive elements was tested with GenometriCorr [48]. The genomic coordinates of non-B DNA conformations were obtained from non-B DB [88].

### 4.6. Analysis of Chromosomal Translocations by In Situ Hi-C

Genome-wide probability of spatial proximity of chromosomal segments was determined by means of in situ Hi-C following the protocol developed by Rao and colleagues [89] using one million cells of HaCaT and HaCaT/SM. Hi-C libraries were paired-end sequenced (2 × 75 bp) on a NextSeq500 (Illumina, San Diego, CA, USA). The software package Juicer was employed for alignment and data analysis using the default setting [90]. Translocation breakpoints were defined by visual inspection of the Hi-C interaction matrices as described previously [42] employing the webtool Juicebox [43] and Circos [46].

### 4.7. Genomic Distribution of Variants and Mutational Signatures

For visualizing the distribution of SNVs across the genome, the Bedtools *map* command was used to sum the number of SNVs per 100 kb genomic bin proceeding from three bedGraph files listing the chromosomal positions of variants both shared and unique to HaCaT and HaCaT/SM (Bedtools v2.17.0; [91]). Results were plotted as heatmaps in the Integrated Genomics Viewer (IGV 2.3.88; [84]). Many of those SNVs identified as unique in either HaCaT or HaCaT/SM are not a consequence of SM treatment, but simply “unique” due to alterations in DNA copy number state, e.g., loss of one chromosome in only one of the two HaCaT cell populations. Therefore, we limited our search for possible SM-related mutational signatures to SNVs mapping to eight chromosomal regions that neither showed any chromosomal differences between HaCaT and HaCaT/SM nor any apparent copy number-associated regional enrichment of SNVs as determined by visual inspection. SNVs mapping to these eight regions, which encompassed 405 Mb of the genome, were extracted from the genome-wide vcf files using the filtering options provided by VCFtools [78]. The resulting 24 vcf files separately representing unique and common SNVs in each of the eight regions were imported into *Mutational Patterns* [54] for further analysis. We followed the workflow described in the vignette of this R package to determine the relative contribution of the six possible base substitution types, to generate 96 trinucleotide count matrices, to extract mutational signatures utilizing non-negative matrix factorization (factorization rank *n* = 3), and to compare our SNV sets to previously published COSMIC mutational signatures (https://cancer.sanger.ac.uk/cosmic/signatures_v2) [50,92,93].

## Data Availability

Hi-C and array CGH data discussed in this publication were deposited in NCBI’s Gene Expression Omnibus (GEO) [94,95] and are accessible through GEO Series accession number GSE162646. Sequencing data are available upon request.

## Supplementary Materials

The following are available online at https://www.mdpi.com/1422-0067/22/3/1146/s1: Figure S1. Chromosomal translocation breakpoints can be inferred from abrupt changes in inter-chromosomal interaction frequencies; Figure S2. Overrepresentation of sequence motifs; Figure S3. DNA copy number exerts influence on the distribution of unique SNVs; Figure S4. Strand-specificity of genic mutations; Table S1. DNA content in HaCaT and HaCaT/SM; Table S2. Genomic coordinates of chromosomal translocations; Table S3. Array CGH results; Table S4. Coordinates of 75 chromosomal breakpoints at base resolution; Table S5. Read statistics of whole-genome sequencing.
