# Supplementary material for: Genomic Adaption and Mutational Patterns in a HaCaT Subline Resistant to Alkylating Agents and Ionizing Radiation
Source: Int J Mol Sci. 2021 Jan 24;22(3):1146. doi: 10.3390/ijms22031146 (PMC7865644; doi:10.3390/ijms22031146)
Supplement: Supplementary file 1 [file ijms-22-01146-s001.zip › Supplementary Tables and Figures.docx]

Figure S1: Chromosomal translocation breakpoints can be inferred from abrupt changes in inter-chromosomal interaction frequencies


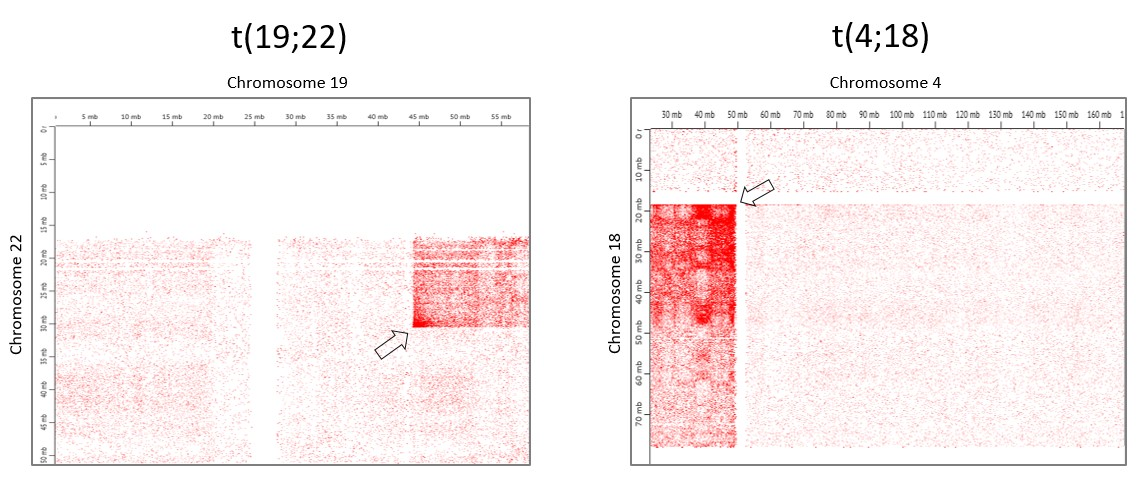


Figure S1:   Chromosomal translocation breakpoints can be inferred from abrupt changes in inter-chromosomal interaction frequencies**.** Pairwise display of translocation partner chromosomes t(19;22) and t(4;18). Red color saturation corresponds to the number of Hi-C interactions between chromosomal regions and hence their probability of spatial proximity within the nucleus. Arrows point to the translocation breakpoints. Note that for translocation t(4;18) both breakpoints are centromeric. At the centromere no chromosomal interactions are displayed due to the lack of mappable reads. Juicebox was employed for visualization of Hi-C data [43].

Figure S2: Overrepresentation of sequence motifs


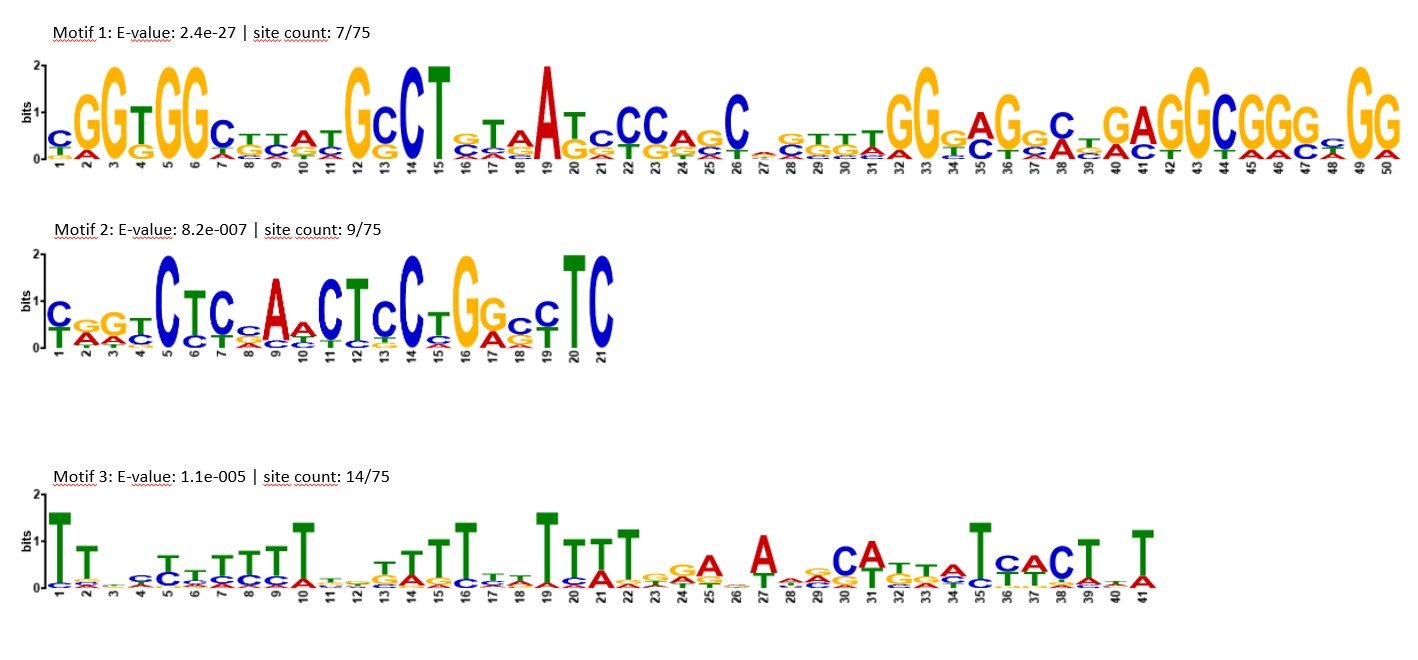


Figure S2: Overrepresentation of sequence motifs detected in a 100bp interval surrounding 75 HaCaT/SM specific chromosomal breakpoints. Sequence intervals containing motif1 and motif2 mapped to repetitive elements (SINEs and LINEs). In all cases, motifs had variable distance to the chromosomal breakpoint.

Figure S3: DNA copy number exerts influence on the distribution of unique SNVs


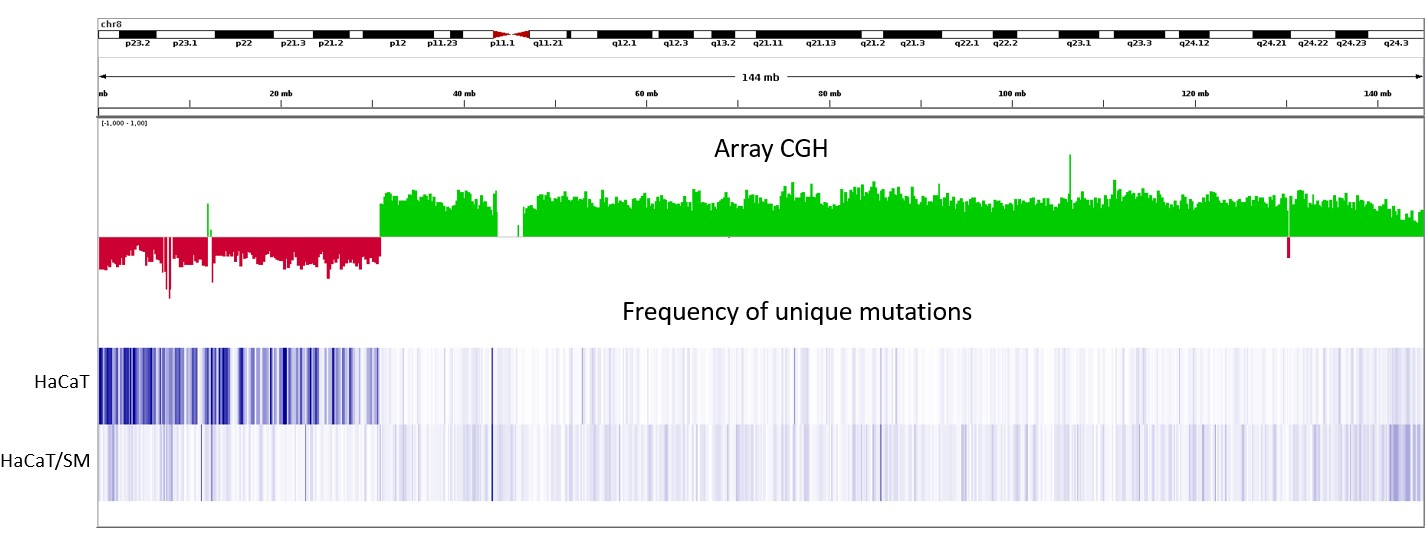


Figure S3: DNA copy number exerts influence on the distribution of unique SNVs. DNA copy number differences between HaCaT and HaCaT/SM are plotted below the chromosome ideogram. Green and red indicate higher and lower DNA copy number in HaCaT/SM, respectively, when compared to HaCaT. The heatmap at the bottom depicts the genomic distribution of unique SNVs for both cell lines. Blue color saturation refers to the normalized frequency of SNVs calculated at a resolution of 100kb.

Figure S4: Strand-specificity of genic mutations


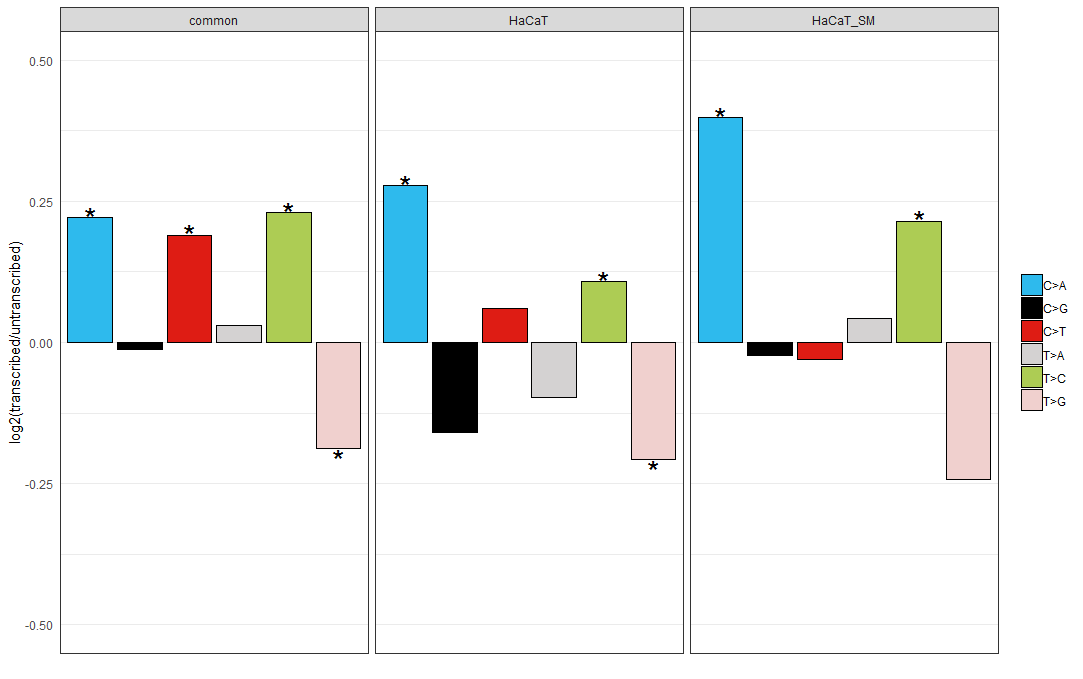


Figure S4: Strand-specificity of genic mutations. The bars represent the log2ratio (transcribed/untranscribed) for each mutation type with reference to the pyrimidine of the DNA double strand. Color legend is provided to the right. Asterisks indicate p<0.05.

**Supplementary Table 1: DNA content in HaCaT and HaCaT/SM**

| **biorep** | **tecrep** | **cell_line** | **ng/µl; 100µl total** |
| --- | --- | --- | --- |
| 1 | 1 | HaCaT | 332 |
| 2 | 1 | HaCaT | 496 |
| 3 | 1 | HaCaT | 270 |
| 1 | 2 | HaCaT | 339 |
| 2 | 2 | HaCaT | 529,3 |
| 3 | 2 | HaCaT | 272,2 |
|  |  | **Average:** | **373** |
| 1 | 1 | HaCaT/SM | 248,4 |
| 2 | 1 | HaCaT/SM | 270 |
| 3 | 1 | HaCaT/SM | 232,7 |
| 1 | 2 | HaCaT/SM | 251,6 |
| 2 | 2 | HaCaT/SM | 253,5 |
| 3 | 2 | HaCaT/SM | 171,4 |
|  |  | **Average:** | **238** |
|  |  |  |  |
|  | biorep: biological replicate |  |  |
|  | tecrep: technical replicate |  |  |

**Supplementary Table 2: Genomic coordinates of chromosomal translocations detected in HaCaT/SM**

| \| Genomic coordinates refer to the 1kb genomic bin in which the interchromosomal Hi-C interactions start (hg19) \| \| \| \| \| \| \| \| --- \| --- \| --- \| --- \| --- \| --- \| --- \| \|  \|  \|  \|  \|  \|  \|  \| \| t(1;7) \| chr1 \| 2783000 \| 2784000 \| chr7 \| 7008000 \| 7009000 \| \| t(1;16) \| chr1 \| 149820000 \| 149821000 \| chr16 \| 70472000 \| 70473000 \| \| t(3;4)* \| chr3 \| Centromer** \| \| chr4 \| Centromer** \| \| \| t(4;14) \| chr4 \| Centromer** \| \| chr14 \| 20199000 \| 20200000 \| \| t(4;18)* \| chr4 \| Centromer** \| \| chr18 \| Centromer** \| \| \| t(5;13) \| chr5 \| 178707000 \| 178708000 \| chr13 \| 23680000 \| 23681000 \| \| t(5;16) \| chr5 \| 141088000 \| 141089000 \| chr16 \| 127000 \| 128000 \| \| t(11;X) \| chrx \| 48923000 \| 48924000 \| chr11** \| 104655000 \| 104660000 \| \| t(8;17) \| chr8** \| 56140000 \| 56145000 \| chr17** \| 36350000 \| 36355000 \| \| t(8;19) \| chr8 \| 30762000 \| 30763000 \| chr19 \| 21747000 \| 21748000 \| \| t(12;20) \| chr12 \| 39243000 \| 39244000 \| chr20 \| 32941000 \| 32942000 \| \| t(14;18) \| chr14 \| 64434000 \| 64435000 \| chr18 \| 43288000 \| 43289000 \| \| t(19;22)* \| chr19 \| 44295000 \| 44296000 \| chr22 \| 30547000 \| 30548000 \| \|  \|  \|  \|  \|  \|  \|  \| \| *translocations common to HaCaT and HaCaT/SM \| \| \| \|  \|  \|  \| \| **breakpoints could not be unambiguously defined at 1kb resolution \| \| \| \| \|  \|  \| | | | | | |  | | | |  |  |
| --- | --- | --- | --- | --- | --- | --- | --- | --- | --- | --- | --- | --- | --- | --- | --- | --- | --- | --- | --- | --- | --- | --- | --- | --- | --- | --- | --- | --- | --- | --- | --- | --- | --- | --- | --- | --- | --- | --- | --- | --- | --- | --- | --- | --- | --- | --- | --- | --- | --- | --- | --- | --- | --- | --- | --- | --- | --- | --- | --- | --- | --- | --- | --- | --- | --- | --- | --- | --- | --- | --- | --- | --- | --- | --- | --- | --- | --- | --- | --- | --- | --- | --- | --- | --- | --- | --- | --- | --- | --- | --- | --- | --- | --- | --- | --- | --- | --- | --- | --- | --- | --- | --- | --- | --- | --- | --- | --- | --- | --- | --- | --- | --- | --- | --- | --- | --- | --- | --- | --- | --- | --- | --- | --- | --- | --- | --- | --- | --- | --- | --- | --- | --- | --- | --- | --- | --- | --- |
|  | | | | | | | | | | | |
|  |  |  |  |  | |  | | | |  |  |
|  |  |  |  |  | |  | | | |  |  |
|  |  |  |  |  | |  | | | |  |  |
|  |  |  | |  | | |  | | | |  |
|  |  |  | |  | |  | | | |  |  |
|  |  |  | | |  | | |  |  |  |  |
|  |  |  |  |  | |  | | | |  |  |
|  |  |  |  |  | |  | | | |  |  |
|  |  |  |  |  | |  | | | |  |  |
|  |  |  |  |  | |  | | | |  |  |
|  |  |  |  |  | |  | | | |  |  |
|  |  |  |  |  | |  | | | |  |  |
|  |  |  |  |  | |  | | | |  |  |

**Supplementary Table 3a: CBS Segments of array CGH data HaCaT_vs_12878**

Chromosome coordinates refer to hg19

| **chromosome** | **start** | **stop** | **average ratio/CBS segment** | **oligos/segment** |
| --- | --- | --- | --- | --- |
| 1 | 564424 | 6976647 | -0,1233 | 930 |
| 1 | 6982100 | 7087071 | 0,8236 | 21 |
| 1 | 7096384 | 55085250 | -0,1371 | 8190 |
| 1 | 55092291 | 55093357 | 3,9575 | 2 |
| 1 | 55103579 | 72768914 | -0,14 | 2897 |
| 1 | 72773396 | 72795480 | 5,0945 | 4 |
| 1 | 72817094 | 145398568 | -0,1672 | 6771 |
| 1 | 145401438 | 208400878 | 0,1573 | 9744 |
| 1 | 208406616 | 209525523 | 0,3451 | 88 |
| 1 | 209542584 | 209635359 | 1,0664 | 25 |
| 1 | 209645905 | 241680610 | 0,2691 | 4982 |
| 1 | 241685334 | 244027216 | 0,1692 | 383 |
| 1 | 244033798 | 244033857 | -0,5632 | 5 |
| 1 | 244042848 | 245634397 | 0,2302 | 274 |
| 1 | 245642873 | 245647443 | 1,421 | 2 |
| 1 | 245653516 | 248158998 | 0,2316 | 489 |
| 1 | 248165114 | 248727988 | 0,0371 | 88 |
| 1 | 248738898 | 248808452 | 0,9667 | 14 |
| 1 | 248811782 | 249218792 | 0,1546 | 49 |
| 2 | 17019 | 89160192 | -0,1375 | 12995 |
| 2 | 89163862 | 89230801 | 4,8709 | 14 |
| 2 | 89234600 | 89312590 | 3,0436 | 8 |
| 2 | 89429843 | 117959715 | -0,1205 | 2880 |
| 2 | 117979245 | 118111203 | -1,53 | 7 |
| 2 | 118137847 | 118207105 | -0,2267 | 7 |
| 2 | 118249154 | 118422291 | -1,5068 | 14 |
| 2 | 118440661 | 243041364 | -0,1329 | 17543 |
| 3 | 62075 | 7766160 | -0,762 | 1147 |
| 3 | 7770630 | 14720384 | -0,6112 | 1143 |
| 3 | 14723757 | 24209826 | -0,7271 | 1305 |
| 3 | 24215000 | 53513704 | -0,6384 | 4567 |
| 3 | 53517908 | 90309067 | -0,7094 | 4974 |
| 3 | 93538467 | 162504822 | 0,1936 | 9888 |
| 3 | 162514534 | 162619141 | -0,8096 | 14 |
| 3 | 162630179 | 192870680 | 0,2064 | 4294 |
| 3 | 192876188 | 192879637 | 4,9575 | 2 |
| 3 | 192885451 | 195354183 | 0,1667 | 471 |
| 3 | 195419168 | 195456540 | -0,644 | 12 |
| 3 | 195457709 | 197845254 | 0,2221 | 430 |
| 4 | 45882 | 3521789 | -0,6182 | 620 |
| 4 | 3527157 | 3580319 | 0,4256 | 9 |
| 4 | 3584553 | 3631805 | 0,0088 | 9 |
| 4 | 3636834 | 7692707 | -0,6696 | 661 |
| 4 | 7700290 | 9738793 | -0,4977 | 262 |
| 4 | 9766686 | 28967318 | -0,6601 | 2246 |
| 4 | 28992834 | 29029856 | -4,501 | 3 |
| 4 | 29042108 | 34757451 | -0,6401 | 397 |
| 4 | 34787128 | 34814779 | -3,886 | 3 |
| 4 | 34833500 | 69438240 | -0,697 | 3819 |
| 4 | 69456442 | 69483277 | -4,0062 | 4 |
| 4 | 69608683 | 173422584 | -0,6889 | 13548 |
| 4 | 173426479 | 173429556 | -4,6213 | 6 |
| 4 | 173436383 | 184601746 | -0,6996 | 1290 |
| 4 | 184605094 | 187268079 | -0,8161 | 468 |
| 4 | 187275868 | 190916678 | -0,6674 | 482 |
| 5 | 26142 | 144838919 | -0,1539 | 17886 |
| 5 | 144852491 | 147039519 | -0,05 | 362 |
| 5 | 147049557 | 147218391 | 0,266 | 35 |
| 5 | 147223546 | 148797991 | -0,0471 | 287 |
| 5 | 148801512 | 148892837 | 0,3693 | 26 |
| 5 | 148898275 | 180712263 | -0,1596 | 4552 |
| 6 | 154130 | 291594 | -0,0662 | 23 |
| 6 | 299363 | 378956 | -0,9451 | 18 |
| 6 | 384100 | 451727 | -0,5207 | 13 |
| 6 | 459017 | 4356111 | -0,1487 | 662 |
| 6 | 4365687 | 4431844 | 0,6504 | 11 |
| 6 | 4437821 | 12970329 | -0,2106 | 1250 |
| 6 | 12974593 | 13232686 | -0,4596 | 59 |
| 6 | 13235912 | 17001496 | -0,2525 | 514 |
| 6 | 17013088 | 23021549 | -0,1293 | 817 |
| 6 | 23035413 | 29833846 | -0,211 | 1159 |
| 6 | 29842826 | 29902314 | 0,8977 | 10 |
| 6 | 29913929 | 29954988 | 0,3516 | 8 |
| 6 | 29961389 | 32450744 | -0,1132 | 497 |
| 6 | 32455274 | 32521929 | 2,1782 | 10 |
| 6 | 32526999 | 170911240 | -0,1921 | 19177 |
| 7 | 44944 | 57593744 | 0,5391 | 8173 |
| 7 | 57621360 | 159118566 | -0,1155 | 16162 |
| 8 | 161472 | 39214817 | 0,0021 | 5568 |
| 8 | 39222367 | 39386158 | -0,5813 | 30 |
| 8 | 39392475 | 52042668 | -0,0127 | 1149 |
| 8 | 52060452 | 55854108 | 0,6529 | 498 |
| 8 | 55864647 | 86842254 | -0,0213 | 3853 |
| 8 | 86847986 | 88361167 | 0,6884 | 253 |
| 8 | 88365946 | 146294098 | 0,0139 | 7880 |
| 9 | 204193 | 23348594 | -0,7161 | 3406 |
| 9 | 23363267 | 23375985 | -3,4005 | 2 |
| 9 | 23387660 | 43686974 | -0,6495 | 2052 |
| 9 | 43704910 | 141025921 | 0,684 | 10856 |
| 10 | 136361 | 135434178 | 0,1212 | 19304 |
| 11 | 200215 | 134945165 | 0,2628 | 19535 |
| 12 | 87496 | 9624859 | -0,0201 | 1638 |
| 12 | 9637323 | 9691488 | 3,748 | 7 |
| 12 | 9698458 | 34371623 | -0,0095 | 3664 |
| 12 | 34382906 | 46350373 | -0,1979 | 1127 |
| 12 | 46354274 | 80618958 | -0,111 | 5226 |
| 12 | 80624821 | 84105922 | -0,2182 | 472 |
| 12 | 84131852 | 88292480 | -0,1184 | 434 |
| 12 | 88326050 | 89831174 | -0,0099 | 160 |
| 12 | 89833852 | 128756577 | -0,1522 | 5834 |
| 12 | 128774220 | 129611356 | -0,4321 | 133 |
| 12 | 129616497 | 133779076 | -0,1453 | 749 |
| 13 | 19296544 | 101251312 | 0,1723 | 11592 |
| 13 | 101255898 | 110941017 | 0,2939 | 1349 |
| 13 | 110945698 | 115107245 | 0,2107 | 713 |
| 14 | 19376762 | 20420849 | -0,6255 | 35 |
| 14 | 20427183 | 106327993 | 0,1659 | 13552 |
| 14 | 106331956 | 106990336 | 3,118 | 92 |
| 14 | 106994552 | 107032628 | 0,8381 | 7 |
| 14 | 107038150 | 107287505 | 0,0507 | 55 |
| 15 | 20102541 | 35537222 | 0,1769 | 1869 |
| 15 | 35541342 | 37726781 | 0,5908 | 290 |
| 15 | 37739875 | 39135277 | 0,4193 | 174 |
| 15 | 39152068 | 40295915 | 0,5993 | 169 |
| 15 | 40300392 | 70605020 | 0,4593 | 5647 |
| 15 | 70616224 | 73846909 | 0,5199 | 603 |
| 15 | 73850337 | 74315103 | 0,7146 | 90 |
| 15 | 74320009 | 76883762 | 0,4546 | 514 |
| 15 | 76893283 | 76893443 | -2,7135 | 2 |
| 15 | 76909072 | 78863764 | 0,3439 | 352 |
| 15 | 78869164 | 80793401 | 0,4318 | 330 |
| 15 | 80796676 | 81030306 | 0,7233 | 44 |
| 15 | 81033125 | 87077646 | 0,4677 | 922 |
| 15 | 87080426 | 87813437 | 0,6303 | 122 |
| 15 | 87833533 | 87865813 | -0,5843 | 3 |
| 15 | 87891864 | 98417331 | 0,4846 | 1628 |
| 15 | 98421197 | 98844626 | 0,6757 | 69 |
| 15 | 98855032 | 101004278 | 0,4125 | 412 |
| 15 | 101009658 | 101621069 | 0,587 | 116 |
| 15 | 101622610 | 102480888 | 0,4321 | 164 |
| 16 | 93628 | 5496388 | 0,151 | 1001 |
| 16 | 5506122 | 13747719 | 0,0721 | 1244 |
| 16 | 13755527 | 21119750 | 0,1431 | 883 |
| 16 | 21122909 | 31955135 | 0,0722 | 1525 |
| 16 | 32471625 | 78366402 | 0,1867 | 4531 |
| 16 | 78372097 | 78381281 | 5,0917 | 3 |
| 16 | 78387160 | 81980285 | 0,1962 | 512 |
| 16 | 81983434 | 82391779 | 0,7579 | 57 |
| 16 | 82415299 | 90163114 | 0,204 | 1312 |
| 17 | 47546 | 8405133 | 0,2025 | 1593 |
| 17 | 8408182 | 11735466 | 0,0889 | 594 |
| 17 | 11740070 | 21179365 | 0,1663 | 1367 |
| 17 | 21182748 | 30322644 | 0,1058 | 971 |
| 17 | 30326899 | 32483944 | -0,0621 | 413 |
| 17 | 32487839 | 36593257 | 0,1455 | 584 |
| 17 | 36600347 | 40881014 | 0,3151 | 843 |
| 17 | 40887394 | 40904742 | 2,2632 | 5 |
| 17 | 40910621 | 44210822 | -0,0768 | 569 |
| 17 | 44214735 | 44351152 | 0,6484 | 17 |
| 17 | 44787180 | 46300855 | -0,099 | 289 |
| 17 | 46304137 | 47895197 | -0,1984 | 313 |
| 17 | 47898791 | 52161196 | -0,0602 | 520 |
| 17 | 52189051 | 67831905 | -0,1477 | 2556 |
| 17 | 67844904 | 81108062 | -0,0749 | 2137 |
| 18 | 14316 | 124700 | -0,2527 | 3 |
| 18 | 131700 | 15072794 | -0,915 | 2211 |
| 18 | 18529851 | 78010032 | 0,0998 | 7677 |
| 19 | 259395 | 28419244 | -0,129 | 4522 |
| 19 | 28431784 | 29977767 | 0,1528 | 166 |
| 19 | 29982900 | 31382941 | -0,2447 | 180 |
| 19 | 31405968 | 35690925 | -0,6159 | 613 |
| 19 | 35694764 | 35699183 | 0,0805 | 6 |
| 19 | 35703456 | 35847245 | -0,6159 | 28 |
| 19 | 35852152 | 35861544 | -1,6703 | 3 |
| 19 | 35864365 | 38698864 | -0,6397 | 513 |
| 19 | 38701797 | 41939551 | -0,5294 | 608 |
| 19 | 41945337 | 42129093 | -0,8602 | 29 |
| 19 | 42132198 | 44290884 | -0,529 | 312 |
| 19 | 44295174 | 58032463 | -0,1126 | 2764 |
| 19 | 58032895 | 58034943 | -1,5965 | 2 |
| 19 | 58039812 | 59095418 | -0,1292 | 221 |
| 20 | 67778 | 1558438 | 0,504 | 257 |
| 20 | 1563715 | 1577359 | -2,8515 | 4 |
| 20 | 1580899 | 5432786 | 0,4916 | 711 |
| 20 | 5432768 | 5921846 | 0,3231 | 82 |
| 20 | 5925489 | 7845618 | 0,4993 | 184 |
| 20 | 7850691 | 10213446 | 0,3721 | 400 |
| 20 | 10219835 | 26182157 | 0,4774 | 2279 |
| 20 | 26186206 | 29633811 | -0,1874 | 14 |
| 20 | 29638711 | 62949149 | 0,4537 | 5385 |
| 21 | 9832448 | 11094138 | 0,1578 | 32 |
| 21 | 11114432 | 16042960 | -0,4069 | 173 |
| 21 | 16048159 | 25839496 | -0,2663 | 1333 |
| 21 | 25842188 | 27264931 | -0,4199 | 205 |
| 21 | 27269816 | 48098603 | -0,2428 | 4090 |
| 22 | 16133474 | 17283881 | -0,0079 | 27 |
| 22 | 17290275 | 24337667 | 0,1923 | 1315 |
| 22 | 24347959 | 24395353 | -0,5714 | 10 |
| 22 | 24405241 | 25552998 | 0,2303 | 240 |
| 22 | 25555820 | 27210464 | 0,1037 | 406 |
| 22 | 27213070 | 30565521 | 0,2229 | 756 |
| 22 | 30568119 | 51224252 | -0,1669 | 4364 |
| X | 61091 | 2701332 | -0,3781 | 779 |
| X | 2709027 | 73988840 | 0,2336 | 9414 |
| X | 73990442 | 97515706 | 0,1591 | 2042 |
| X | 97529082 | 154929332 | 0,243 | 7961 |
| X | 154933496 | 155257126 | -0,3803 | 93 |
| Y | 2650450 | 9905743 | -2,6625 | 465 |
| Y | 9925786 | 13213533 | -0,378 | 12 |
| Y | 13942734 | 27328334 | -2,6852 | 994 |
| Y | 28460973 | 59031480 | -1,0602 | 48 |

**Supplementary Table 3b: CBS Segments of array CGH data HaCaT/SM_vs_12878**

| **chromosome** | **start** | **stop** | **average ratio/CBS segment** | **oligos/segment** |
| --- | --- | --- | --- | --- |
| 1 | 564424 | 2765252 | -0,9808 | 314 |
| 1 | 2771708 | 6976647 | -0,1992 | 616 |
| 1 | 6982100 | 7101811 | 0,6817 | 23 |
| 1 | 7105853 | 55085250 | -0,1941 | 8188 |
| 1 | 55092291 | 55093357 | 3,546 | 2 |
| 1 | 55103579 | 72768914 | -0,1605 | 2897 |
| 1 | 72773396 | 72795480 | 4,8152 | 4 |
| 1 | 72817094 | 74808558 | -0,1549 | 173 |
| 1 | 74818957 | 76455553 | -0,2477 | 256 |
| 1 | 76478240 | 76890734 | -0,3679 | 79 |
| 1 | 76893876 | 79680637 | -0,23 | 460 |
| 1 | 79697133 | 86186954 | -0,1796 | 770 |
| 1 | 86191988 | 86195086 | 1,2505 | 2 |
| 1 | 86199985 | 94461769 | -0,1907 | 1276 |
| 1 | 94465555 | 95015887 | -0,0334 | 103 |
| 1 | 95019423 | 95028972 | 0,3578 | 6 |
| 1 | 95048829 | 96808710 | -0,0653 | 212 |
| 1 | 96819419 | 97994780 | -0,1912 | 186 |
| 1 | 98000984 | 99223505 | -0,0625 | 182 |
| 1 | 99230386 | 102579999 | -0,2555 | 482 |
| 1 | 102595342 | 106497352 | -0,1492 | 283 |
| 1 | 106508508 | 108900263 | -0,2497 | 286 |
| 1 | 108926313 | 108968593 | 2,0545 | 2 |
| 1 | 109014397 | 111661485 | -0,1937 | 484 |
| 1 | 111665143 | 112471715 | -0,3209 | 163 |
| 1 | 112478576 | 115289544 | -0,2047 | 447 |
| 1 | 115292392 | 116866167 | -0,1008 | 222 |
| 1 | 116889082 | 149063254 | -0,1978 | 959 |
| 1 | 149079747 | 249218792 | 0,1846 | 15882 |
| 2 | 17019 | 446897 | -0,138 | 62 |
| 2 | 453318 | 8779759 | -0,2565 | 1040 |
| 2 | 8789269 | 22905809 | -0,1822 | 1679 |
| 2 | 22922433 | 23274286 | -0,2975 | 28 |
| 2 | 23290112 | 23875155 | -0,4186 | 60 |
| 2 | 23880439 | 24516594 | -0,2627 | 125 |
| 2 | 24520261 | 24544287 | -1,1344 | 5 |
| 2 | 24548386 | 25745133 | -0,3057 | 228 |
| 2 | 25749583 | 27656313 | -0,2185 | 378 |
| 2 | 27659628 | 29177071 | -0,0774 | 297 |
| 2 | 29180808 | 33062794 | -0,2297 | 699 |
| 2 | 33068816 | 34303636 | -0,3142 | 238 |
| 2 | 34311133 | 34688779 | -0,2131 | 76 |
| 2 | 34697718 | 34730142 | -1,057 | 7 |
| 2 | 34738177 | 59738672 | -0,1771 | 3993 |
| 2 | 59749446 | 60957888 | -0,3277 | 133 |
| 2 | 60961650 | 70783525 | -0,2065 | 1530 |
| 2 | 70788847 | 70903393 | 0,1476 | 17 |
| 2 | 70912001 | 71955960 | -0,2173 | 185 |
| 2 | 71970872 | 73142474 | -0,0913 | 178 |
| 2 | 73146637 | 74503741 | -0,2381 | 256 |
| 2 | 74508883 | 74576618 | 0,1999 | 15 |
| 2 | 74580924 | 74918152 | -0,2552 | 76 |
| 2 | 74924843 | 78782131 | -0,1655 | 492 |
| 2 | 78787522 | 81509992 | -0,258 | 377 |
| 2 | 81509933 | 84801042 | -0,124 | 244 |
| 2 | 84804466 | 89160192 | -0,2402 | 577 |
| 2 | 89163862 | 89312590 | 4,0298 | 22 |
| 2 | 89429843 | 101233882 | -0,2282 | 905 |
| 2 | 101238822 | 102979229 | -0,093 | 294 |
| 2 | 102984488 | 117959715 | -0,1879 | 1681 |
| 2 | 117979245 | 118111203 | -1,1773 | 7 |
| 2 | 118137847 | 118207105 | -0,3033 | 7 |
| 2 | 118249154 | 118398138 | -1,1835 | 13 |
| 2 | 118422232 | 144743633 | -0,203 | 3310 |
| 2 | 144751679 | 144838455 | -1,1101 | 17 |
| 2 | 144844175 | 243041364 | -0,1919 | 14216 |
| 3 | 62075 | 2237271 | -0,1573 | 325 |
| 3 | 2240480 | 4759473 | -0,2889 | 441 |
| 3 | 4765018 | 8460961 | -0,1548 | 448 |
| 3 | 8465947 | 9048409 | -1,0118 | 92 |
| 3 | 9057345 | 60360280 | -0,2097 | 8005 |
| 3 | 60367507 | 60416652 | -1,0248 | 11 |
| 3 | 60422896 | 60544760 | -3,8019 | 25 |
| 3 | 60551364 | 90291702 | -0,2115 | 3788 |
| 3 | 90309008 | 106901887 | 0,3484 | 1590 |
| 3 | 106912804 | 107368692 | -0,2336 | 69 |
| 3 | 107372962 | 168856129 | 0,3283 | 8912 |
| 3 | 168863349 | 169365805 | -0,1341 | 103 |
| 3 | 169371820 | 169798948 | 0,2971 | 84 |
| 3 | 169803088 | 172525668 | 0,363 | 482 |
| 3 | 172528512 | 172538403 | -0,2173 | 6 |
| 3 | 172545535 | 177915132 | 0,3822 | 712 |
| 3 | 177940982 | 179993342 | 0,2496 | 318 |
| 3 | 180010415 | 180511081 | 0,6216 | 67 |
| 3 | 180521519 | 188806609 | 0,3397 | 1295 |
| 3 | 188819304 | 189588477 | 0,4883 | 121 |
| 3 | 189592668 | 189684755 | 1,7323 | 18 |
| 3 | 189688623 | 192870680 | 0,3438 | 420 |
| 3 | 192876188 | 192879637 | 5,002 | 2 |
| 3 | 192885451 | 193403951 | 0,3389 | 99 |
| 3 | 193407686 | 193413976 | -0,5455 | 2 |
| 3 | 193417409 | 194400680 | 0,3786 | 167 |
| 3 | 194406140 | 195354183 | 0,2674 | 203 |
| 3 | 195419168 | 195454513 | -0,5325 | 11 |
| 3 | 195456487 | 195546181 | 0,1046 | 17 |
| 3 | 195553573 | 195585866 | 0,5027 | 7 |
| 3 | 195591215 | 195740416 | 0,084 | 18 |
| 3 | 195747856 | 196787620 | 0,3193 | 203 |
| 3 | 196793590 | 197167830 | 0,4815 | 65 |
| 3 | 197177464 | 197845254 | 0,2895 | 121 |
| 4 | 45882 | 3535755 | -0,2906 | 623 |
| 4 | 3574456 | 3631805 | 0,1862 | 15 |
| 4 | 3636834 | 7671306 | -0,3241 | 655 |
| 4 | 7675365 | 8269985 | -0,1471 | 127 |
| 4 | 8276386 | 11346318 | -0,2904 | 319 |
| 4 | 11349651 | 16609794 | -0,1837 | 611 |
| 4 | 16619420 | 19085914 | -0,2813 | 284 |
| 4 | 19100354 | 25238612 | -0,1548 | 788 |
| 4 | 25239992 | 27178520 | -0,323 | 269 |
| 4 | 27190115 | 28967318 | -0,1763 | 116 |
| 4 | 28992834 | 29029856 | -4,561 | 3 |
| 4 | 29042108 | 34757451 | -0,15 | 397 |
| 4 | 34787128 | 34814779 | -4,538 | 3 |
| 4 | 34833500 | 69387115 | -0,2134 | 3812 |
| 4 | 69392545 | 69483277 | -2,3892 | 11 |
| 4 | 69608683 | 70985075 | -0,1092 | 171 |
| 4 | 70994735 | 73979545 | -0,2524 | 427 |
| 4 | 73984370 | 75772429 | -0,0845 | 252 |
| 4 | 75792055 | 77920891 | -0,2065 | 352 |
| 4 | 77924761 | 78639120 | -0,1183 | 125 |
| 4 | 78647247 | 80402284 | 0,3208 | 287 |
| 4 | 80418371 | 87247773 | -0,1813 | 1015 |
| 4 | 87253172 | 87514551 | -1,125 | 44 |
| 4 | 87519718 | 173426538 | -0,1834 | 10876 |
| 4 | 173429497 | 173429556 | -5,5516 | 5 |
| 4 | 173436383 | 175187359 | -0,2001 | 266 |
| 4 | 175192070 | 175981630 | -0,0413 | 107 |
| 4 | 176006233 | 177414321 | -0,2197 | 182 |
| 4 | 177429071 | 179975909 | -0,1023 | 243 |
| 4 | 179989088 | 184618727 | -0,2028 | 498 |
| 4 | 184622595 | 187268079 | -0,3102 | 462 |
| 4 | 187275868 | 190519828 | -0,1273 | 438 |
| 4 | 190530860 | 190916678 | -0,3285 | 44 |
| 5 | 26142 | 226417 | 0,7068 | 30 |
| 5 | 241803 | 7634436 | 0,2648 | 964 |
| 5 | 7639686 | 8582426 | -0,2973 | 112 |
| 5 | 8597424 | 11456292 | 0,2862 | 436 |
| 5 | 11460177 | 11460236 | -1,5972 | 5 |
| 5 | 11465346 | 11673245 | 0,2627 | 42 |
| 5 | 11679299 | 13944218 | 0,4066 | 195 |
| 5 | 13951956 | 16841573 | 0,2871 | 450 |
| 5 | 16845129 | 31885967 | 0,3897 | 1426 |
| 5 | 31891932 | 36072370 | 0,2282 | 649 |
| 5 | 36081736 | 40410866 | 0,3495 | 584 |
| 5 | 40441340 | 45972996 | 0,2826 | 740 |
| 5 | 46100308 | 59481493 | -0,1682 | 1315 |
| 5 | 59493142 | 59799206 | 0,257 | 42 |
| 5 | 59806227 | 87548782 | -0,2012 | 3551 |
| 5 | 87552335 | 89534065 | -0,3775 | 187 |
| 5 | 89544201 | 96269371 | -0,1852 | 963 |
| 5 | 96273265 | 102191620 | -0,2538 | 548 |
| 5 | 102198168 | 106902674 | -0,1682 | 424 |
| 5 | 106907761 | 109702502 | -0,2886 | 404 |
| 5 | 109708245 | 130093367 | -0,1951 | 2454 |
| 5 | 130113270 | 130220806 | 0,8865 | 6 |
| 5 | 130242760 | 134792981 | -0,2083 | 802 |
| 5 | 134797172 | 136460806 | -0,096 | 230 |
| 5 | 136466671 | 139741563 | -0,2605 | 599 |
| 5 | 139744099 | 141076783 | -0,1549 | 269 |
| 5 | 141082661 | 178706458 | 0,2959 | 5372 |
| 5 | 178711746 | 180712263 | -0,2505 | 349 |
| 6 | 154130 | 255409 | -0,3818 | 16 |
| 6 | 259881 | 291594 | 0,077 | 7 |
| 6 | 299363 | 378956 | -1,1668 | 18 |
| 6 | 384100 | 780761 | -0,3672 | 70 |
| 6 | 794273 | 3933095 | -0,1744 | 537 |
| 6 | 3939209 | 4008723 | 0,6611 | 17 |
| 6 | 4018128 | 4356111 | -0,1279 | 51 |
| 6 | 4365687 | 4437880 | 0,4446 | 12 |
| 6 | 4450082 | 4781576 | -0,1181 | 55 |
| 6 | 4784693 | 6313643 | -0,2861 | 273 |
| 6 | 6318938 | 7266135 | -0,2421 | 133 |
| 6 | 7269672 | 7792889 | -0,0837 | 92 |
| 6 | 7797658 | 13970236 | -0,2172 | 898 |
| 6 | 13978235 | 17474337 | -0,3054 | 427 |
| 6 | 17479222 | 17597990 | -0,1738 | 25 |
| 6 | 17605080 | 18565451 | -0,0514 | 185 |
| 6 | 18567127 | 20919550 | -0,1652 | 298 |
| 6 | 20922535 | 21040431 | -1,066 | 25 |
| 6 | 21043768 | 29842885 | -0,2296 | 1388 |
| 6 | 29854870 | 29902314 | 0,7056 | 9 |
| 6 | 29913929 | 29954988 | 0,1506 | 8 |
| 6 | 29961389 | 32450744 | -0,2004 | 497 |
| 6 | 32455274 | 32521929 | 2,3121 | 10 |
| 6 | 32526999 | 112343408 | -0,2006 | 10632 |
| 6 | 112355152 | 118016721 | 0,4017 | 676 |
| 6 | 118024124 | 119962083 | 0,2539 | 299 |
| 6 | 119978573 | 129734142 | 0,3942 | 1411 |
| 6 | 129737392 | 133557506 | 0,2786 | 584 |
| 6 | 133561063 | 143891313 | 0,3264 | 1477 |
| 6 | 143904007 | 166246864 | 0,2635 | 3383 |
| 6 | 166255399 | 169059443 | 0,1834 | 446 |
| 6 | 169062414 | 170911240 | 0,2719 | 269 |
| 7 | 44944 | 183615 | 0,8468 | 26 |
| 7 | 190585 | 1818849 | 0,6195 | 223 |
| 7 | 1828396 | 1832486 | 1,9615 | 2 |
| 7 | 1844367 | 3258015 | 0,6144 | 270 |
| 7 | 3272602 | 3965563 | 0,7518 | 128 |
| 7 | 3969060 | 5662869 | 0,6066 | 284 |
| 7 | 5667980 | 6522389 | 0,7352 | 133 |
| 7 | 6531575 | 6861119 | 0,6198 | 46 |
| 7 | 6870884 | 7753908 | 0,3637 | 110 |
| 7 | 7759658 | 9145132 | 0,2494 | 214 |
| 7 | 9155395 | 11445872 | 0,356 | 231 |
| 7 | 11450032 | 14429304 | 0,4164 | 395 |
| 7 | 14437193 | 18106124 | 0,4938 | 565 |
| 7 | 18125585 | 20766782 | 0,399 | 362 |
| 7 | 20773076 | 78655036 | 0,3135 | 7225 |
| 7 | 78657843 | 85420011 | 0,4756 | 861 |
| 7 | 85434791 | 100324974 | 0,3471 | 2306 |
| 7 | 100333268 | 100337114 | -3,4225 | 2 |
| 7 | 100341655 | 159118566 | 0,3227 | 10954 |
| 8 | 161472 | 30748085 | -0,183 | 4445 |
| 8 | 30775975 | 31497313 | 0,282 | 75 |
| 8 | 31502469 | 32616853 | 0,5696 | 222 |
| 8 | 32621890 | 39226386 | 0,3425 | 827 |
| 8 | 39234992 | 39356881 | -0,1921 | 23 |
| 8 | 39362828 | 39408971 | 0,1899 | 8 |
| 8 | 39416498 | 40440475 | 0,4723 | 130 |
| 8 | 40443459 | 49073857 | 0,2912 | 670 |
| 8 | 49091300 | 50262538 | 0,5419 | 108 |
| 8 | 50268795 | 51024976 | 0,3744 | 81 |
| 8 | 51031424 | 51035969 | 1,2987 | 6 |
| 8 | 51039937 | 52042668 | 0,4019 | 151 |
| 8 | 52060452 | 55854108 | 1,0218 | 498 |
| 8 | 55864647 | 56097345 | 0,292 | 28 |
| 8 | 56101010 | 56147142 | 0,8213 | 10 |
| 8 | 56150183 | 56186639 | 0,4076 | 8 |
| 8 | 56192912 | 57382568 | 0,2142 | 202 |
| 8 | 57386858 | 58878365 | 0,3556 | 136 |
| 8 | 58883606 | 59356817 | 0,186 | 68 |
| 8 | 59360370 | 61197321 | 0,3486 | 200 |
| 8 | 61204542 | 62479746 | 0,2178 | 188 |
| 8 | 62489264 | 63535874 | 0,4667 | 139 |
| 8 | 63541594 | 70373873 | 0,3337 | 951 |
| 8 | 70379159 | 71556437 | 0,2271 | 196 |
| 8 | 71559402 | 81417619 | 0,3335 | 1118 |
| 8 | 81422563 | 86548090 | 0,416 | 608 |
| 8 | 86842195 | 88357734 | 1,111 | 253 |
| 8 | 88361108 | 146294098 | 0,3408 | 7881 |
| 9 | 204193 | 23348594 | -0,1983 | 3406 |
| 9 | 23363267 | 23375985 | -3,752 | 2 |
| 9 | 23387660 | 43686974 | -0,1962 | 2052 |
| 9 | 43704910 | 72001199 | 0,5377 | 195 |
| 9 | 72007275 | 109078408 | 0,675 | 5289 |
| 9 | 109087709 | 109538143 | 0,2792 | 52 |
| 9 | 109564685 | 115420945 | 0,7032 | 955 |
| 9 | 115426736 | 117073702 | 0,587 | 292 |
| 9 | 117079086 | 117086334 | -0,1367 | 3 |
| 9 | 117093018 | 117407976 | 0,5571 | 58 |
| 9 | 117415003 | 119419531 | 0,751 | 295 |
| 9 | 119422945 | 119478493 | -0,0091 | 12 |
| 9 | 119484432 | 124820173 | 0,6816 | 713 |
| 9 | 124828378 | 141025921 | 0,5919 | 2992 |
| 10 | 136361 | 82876632 | -0,195 | 11285 |
| 10 | 82883786 | 82891283 | -5,0665 | 2 |
| 10 | 82897174 | 135434178 | -0,1984 | 8018 |
| 11 | 200215 | 9338441 | 0,2331 | 1687 |
| 11 | 9341652 | 16865856 | 0,3737 | 1169 |
| 11 | 16873547 | 22727016 | 0,2963 | 972 |
| 11 | 22731939 | 31061753 | 0,4132 | 935 |
| 11 | 31068171 | 33277317 | 0,3225 | 371 |
| 11 | 33280396 | 33895328 | 0,2261 | 95 |
| 11 | 33902330 | 33903444 | -0,1834 | 7 |
| 11 | 33908706 | 34139691 | 0,2218 | 29 |
| 11 | 34145871 | 36596011 | 0,3497 | 440 |
| 11 | 36599372 | 43010287 | 0,3898 | 521 |
| 11 | 43031769 | 44249764 | 0,2764 | 199 |
| 11 | 44255643 | 44255702 | -0,672 | 5 |
| 11 | 44262356 | 55834037 | 0,2831 | 958 |
| 11 | 55837305 | 56667870 | 0,4112 | 154 |
| 11 | 56672324 | 66704236 | 0,272 | 1879 |
| 11 | 66712836 | 66718240 | 2,2155 | 2 |
| 11 | 66721519 | 83886757 | 0,2895 | 2600 |
| 11 | 83889549 | 90407290 | 0,351 | 962 |
| 11 | 90418641 | 91116778 | 0,5294 | 80 |
| 11 | 91138165 | 104654343 | 0,3738 | 1780 |
| 11 | 104666540 | 134945165 | -0,2421 | 4689 |
| 12 | 87496 | 5169696 | 0,2618 | 921 |
| 12 | 5175114 | 6909941 | 0,369 | 294 |
| 12 | 6919392 | 9624859 | 0,2533 | 423 |
| 12 | 9637323 | 9691488 | 4,0297 | 7 |
| 12 | 9698458 | 39233768 | 0,3651 | 3758 |
| 12 | 39238668 | 43017521 | 0,6811 | 566 |
| 12 | 43021482 | 43026270 | 1,594 | 2 |
| 12 | 43031056 | 45747247 | 0,7139 | 377 |
| 12 | 45752353 | 46287359 | -0,2351 | 75 |
| 12 | 46290601 | 70820980 | -0,1808 | 3946 |
| 12 | 70824942 | 80293946 | -0,1138 | 1260 |
| 12 | 80298399 | 86281762 | -0,2066 | 702 |
| 12 | 86288447 | 89851649 | -0,0584 | 401 |
| 12 | 89855142 | 91332441 | -0,1924 | 125 |
| 12 | 91337149 | 91621113 | -0,0295 | 55 |
| 12 | 91643465 | 98854294 | -0,1998 | 992 |
| 12 | 98860918 | 98959431 | -0,2577 | 18 |
| 12 | 98964569 | 99000591 | -1,096 | 10 |
| 12 | 99007493 | 128790595 | -0,2336 | 4634 |
| 12 | 128798672 | 129591111 | -0,4359 | 126 |
| 12 | 129595691 | 133779076 | -0,244 | 753 |
| 13 | 19296544 | 19576452 | -0,2782 | 20 |
| 13 | 19583361 | 21013860 | -0,1216 | 229 |
| 13 | 21018101 | 23692245 | -0,2322 | 398 |
| 13 | 23699999 | 54433080 | 0,2999 | 5221 |
| 13 | 54438146 | 110941017 | 0,3723 | 7073 |
| 13 | 110945698 | 115107245 | 0,2738 | 713 |
| 14 | 19376762 | 20427242 | -0,9368 | 36 |
| 14 | 20465947 | 20875310 | -0,1359 | 89 |
| 14 | 20880746 | 64434499 | 0,3184 | 6401 |
| 14 | 64439329 | 64467245 | 1,1463 | 7 |
| 14 | 64469975 | 64711970 | 0,6853 | 65 |
| 14 | 64716278 | 64806824 | 1,0421 | 20 |
| 14 | 64812788 | 67323167 | 0,6442 | 406 |
| 14 | 67333561 | 67449489 | 0,3789 | 23 |
| 14 | 67453057 | 68405559 | 0,6631 | 209 |
| 14 | 68409163 | 68981584 | -0,2289 | 122 |
| 14 | 68985568 | 80244341 | 0,6063 | 2212 |
| 14 | 80249161 | 81753813 | 0,6905 | 252 |
| 14 | 81759152 | 87464269 | 0,7739 | 492 |
| 14 | 87480160 | 89163287 | 0,6778 | 216 |
| 14 | 89168802 | 93592616 | 0,5439 | 858 |
| 14 | 93603795 | 94827068 | 0,6491 | 235 |
| 14 | 94832223 | 98815883 | 0,5312 | 542 |
| 14 | 98819087 | 103831261 | 0,62 | 936 |
| 14 | 103838907 | 103877187 | 1,0542 | 11 |
| 14 | 103881630 | 104508390 | 0,6529 | 135 |
| 14 | 104517193 | 104988578 | 0,4883 | 84 |
| 14 | 104992793 | 105127910 | 0,855 | 27 |
| 14 | 105130812 | 106263147 | 0,5262 | 197 |
| 14 | 106267622 | 106327993 | 1,2797 | 12 |
| 14 | 106331956 | 106990336 | 3,4399 | 92 |
| 14 | 106994552 | 107022520 | 1,4502 | 6 |
| 14 | 107032582 | 107287505 | 0,5375 | 56 |
| 15 | 20102541 | 22107506 | 0,0417 | 52 |
| 15 | 22304596 | 22578630 | 0,4673 | 35 |
| 15 | 22756650 | 29852218 | -0,2511 | 939 |
| 15 | 29856529 | 30276969 | -0,0086 | 74 |
| 15 | 30285843 | 35740179 | -0,2218 | 799 |
| 15 | 35746986 | 37373594 | -0,0694 | 233 |
| 15 | 37376487 | 50389968 | -0,1954 | 2313 |
| 15 | 50399128 | 50427567 | -1,1346 | 8 |
| 15 | 50431866 | 76883762 | -0,2095 | 4903 |
| 15 | 76893283 | 76893443 | -3,9975 | 2 |
| 15 | 76909072 | 80580102 | -0,2693 | 647 |
| 15 | 80589502 | 102480888 | -0,1991 | 3515 |
| 16 | 93628 | 11098095 | 0,2302 | 1827 |
| 16 | 11102191 | 11231125 | -0,3119 | 28 |
| 16 | 11237852 | 33463017 | 0,2107 | 2815 |
| 16 | 33465513 | 33630726 | 0,7194 | 13 |
| 16 | 33651706 | 33961233 | -0,2097 | 6 |
| 16 | 34197413 | 48587596 | 0,2497 | 455 |
| 16 | 48595986 | 51222861 | 0,1375 | 347 |
| 16 | 51231504 | 55314082 | 0,2565 | 440 |
| 16 | 55320395 | 55695483 | 0,4252 | 60 |
| 16 | 55704815 | 59744700 | 0,2349 | 664 |
| 16 | 59750881 | 60358548 | 0,4176 | 61 |
| 16 | 60365279 | 65069453 | 0,2865 | 391 |
| 16 | 65072470 | 66369019 | 0,3664 | 140 |
| 16 | 66375590 | 70503101 | 0,2116 | 821 |
| 16 | 70507381 | 78366402 | -0,229 | 1116 |
| 16 | 78372097 | 78381281 | 3,9343 | 3 |
| 16 | 78387160 | 78459295 | -0,5627 | 15 |
| 16 | 78466362 | 78620872 | -4,0822 | 27 |
| 16 | 78625861 | 79009057 | -1,2181 | 72 |
| 16 | 79015710 | 81991597 | -0,2071 | 404 |
| 16 | 81996980 | 82391779 | 0,3891 | 51 |
| 16 | 82415299 | 82920558 | -0,2014 | 75 |
| 16 | 82926663 | 83463890 | -1,1039 | 108 |
| 16 | 83469809 | 85342231 | -0,1971 | 351 |
| 16 | 85348928 | 86774698 | -0,3107 | 218 |
| 16 | 86784941 | 87889547 | -0,1734 | 179 |
| 16 | 87904877 | 90163114 | 0,2288 | 381 |
| 17 | 47546 | 1952633 | 0,2836 | 378 |
| 17 | 1955398 | 44210822 | -0,2236 | 6561 |
| 17 | 44214735 | 44351152 | 0,7231 | 17 |
| 17 | 44787180 | 46405798 | -0,2002 | 309 |
| 17 | 46410502 | 47892178 | -0,304 | 292 |
| 17 | 47895138 | 52161196 | -0,1499 | 521 |
| 17 | 52189051 | 64788123 | -0,2261 | 2040 |
| 17 | 64789735 | 64854996 | 0,388 | 17 |
| 17 | 64861451 | 67633509 | -0,2292 | 485 |
| 17 | 67647766 | 69086337 | -0,08 | 132 |
| 17 | 69107506 | 69473828 | 0,0893 | 32 |
| 17 | 69497178 | 70870389 | -0,1072 | 162 |
| 17 | 70876723 | 81108062 | -0,2055 | 1825 |
| 18 | 14316 | 124700 | -0,2037 | 3 |
| 18 | 131700 | 14101582 | -1,0932 | 2159 |
| 18 | 14101869 | 15072794 | -0,679 | 52 |
| 18 | 18529851 | 18978578 | 0,4733 | 80 |
| 18 | 18983912 | 21224147 | 0,3432 | 349 |
| 18 | 21229142 | 21776176 | 0,4228 | 108 |
| 18 | 21780630 | 25029649 | 0,2881 | 456 |
| 18 | 25033860 | 29225449 | 0,4424 | 401 |
| 18 | 29229200 | 31659244 | 0,3043 | 402 |
| 18 | 31666325 | 31730206 | 0,7004 | 13 |
| 18 | 31733233 | 34573303 | 0,3373 | 456 |
| 18 | 34576844 | 36540552 | 0,227 | 176 |
| 18 | 36561713 | 39024277 | 0,3709 | 194 |
| 18 | 39045863 | 41206594 | 0,2637 | 249 |
| 18 | 41221713 | 42437315 | 0,428 | 110 |
| 18 | 42441709 | 43301228 | 1,0034 | 129 |
| 18 | 43305236 | 43435785 | 0,7595 | 35 |
| 18 | 43441088 | 43813909 | 0,3743 | 69 |
| 18 | 43818327 | 45933313 | 0,7113 | 315 |
| 18 | 45953441 | 46074744 | 1,2176 | 14 |
| 18 | 46079917 | 47763910 | 0,7219 | 299 |
| 18 | 47769420 | 47802768 | 0,3886 | 7 |
| 18 | 47810513 | 72351388 | -0,1848 | 2977 |
| 18 | 72357349 | 72721380 | -1,1588 | 74 |
| 18 | 72726415 | 78010032 | -0,2605 | 764 |
| 19 | 259395 | 19699438 | -0,218 | 3886 |
| 19 | 19703497 | 21746048 | 0,2395 | 300 |
| 19 | 21749989 | 28302247 | -0,2665 | 326 |
| 19 | 28315299 | 32873003 | -0,3502 | 483 |
| 19 | 32876308 | 35690925 | -0,2654 | 486 |
| 19 | 35694764 | 35694823 | 0,5156 | 5 |
| 19 | 35699124 | 35847245 | -0,3416 | 29 |
| 19 | 35852152 | 35861544 | -1,459 | 3 |
| 19 | 35864365 | 44295233 | -0,2766 | 1463 |
| 19 | 44300485 | 45427674 | 0,272 | 225 |
| 19 | 45431107 | 54168373 | 0,1751 | 1767 |
| 19 | 54170842 | 54393511 | 0,2645 | 77 |
| 19 | 54398787 | 57205023 | 0,5139 | 538 |
| 19 | 57211383 | 57669059 | 1,0382 | 77 |
| 19 | 57671961 | 57775778 | -0,3266 | 22 |
| 19 | 57780334 | 58528678 | 0,1143 | 158 |
| 19 | 58534319 | 58595237 | 0,4455 | 15 |
| 19 | 58599307 | 59095418 | 0,127 | 107 |
| 20 | 67778 | 1558438 | 0,1788 | 257 |
| 20 | 1563715 | 1577359 | -3,51 | 4 |
| 20 | 1580899 | 10494940 | 0,196 | 1435 |
| 20 | 10504045 | 11822205 | 0,3743 | 138 |
| 20 | 11830060 | 18494785 | 0,2222 | 1029 |
| 20 | 18504970 | 18632899 | -0,3275 | 26 |
| 20 | 18639162 | 20257853 | 0,2645 | 262 |
| 20 | 20259807 | 21800424 | 0,1158 | 232 |
| 20 | 21810074 | 22732365 | 0,2363 | 79 |
| 20 | 22738498 | 24473833 | 0,3051 | 237 |
| 20 | 24478450 | 25130054 | 0,1203 | 95 |
| 20 | 25140219 | 26187794 | 0,2584 | 125 |
| 20 | 26191120 | 26213170 | -0,1793 | 10 |
| 20 | 26225123 | 30688718 | 0,2359 | 165 |
| 20 | 30691839 | 32016344 | 0,1133 | 262 |
| 20 | 32020169 | 32037695 | -0,42 | 5 |
| 20 | 32044971 | 32837318 | 0,1959 | 140 |
| 20 | 32842655 | 62949149 | 0,5846 | 4815 |
| 21 | 9832448 | 16269496 | -0,2608 | 244 |
| 21 | 16286146 | 25292082 | -0,1369 | 1252 |
| 21 | 25303376 | 27960660 | -0,2766 | 383 |
| 21 | 27964351 | 30035465 | -0,0709 | 223 |
| 21 | 30043939 | 48098603 | -0,2183 | 3731 |
| 22 | 16133474 | 24337667 | 0,2319 | 1342 |
| 22 | 24347959 | 24390254 | -1,025 | 9 |
| 22 | 24395305 | 25558527 | 0,2662 | 243 |
| 22 | 25561201 | 27205728 | 0,1517 | 403 |
| 22 | 27210405 | 30550232 | 0,2773 | 753 |
| 22 | 30553654 | 48915003 | -0,2354 | 3901 |
| 22 | 48919883 | 49943525 | 0,1786 | 195 |
| 22 | 49948240 | 50546880 | -0,2334 | 122 |
| 22 | 50552774 | 50579476 | 0,0639 | 10 |
| 22 | 50585430 | 51224252 | -0,2487 | 140 |
| X | 61091 | 2698172 | -0,85 | 778 |
| X | 2701273 | 48923131 | -0,1636 | 6755 |
| X | 48928825 | 48942873 | -2,8853 | 4 |
| X | 48949306 | 102623999 | -0,2004 | 5339 |
| X | 102627325 | 108363771 | -0,0921 | 964 |
| X | 108375198 | 109353575 | -0,198 | 138 |
| X | 109358110 | 109515685 | -3,6147 | 33 |
| X | 109522397 | 113161299 | -0,2257 | 538 |
| X | 113166381 | 115926707 | -0,0383 | 388 |
| X | 115944547 | 136328213 | -0,1999 | 2865 |
| X | 136342979 | 154929279 | 0,702 | 2395 |
| X | 154929284 | 155257126 | -0,1802 | 94 |
| Y | 2650450 | 9905743 | -3,0235 | 465 |
| Y | 9925786 | 13942793 | -0,7585 | 13 |
| Y | 13992304 | 27328334 | -3,021 | 993 |
| Y | 28460973 | 59031480 | -1,2008 | 48 |

**Supplementary Table 3c: CBS Segments of array CGH data HaCaT/SM_vs_HaCaT**

Chromosome coordinates refer to hg19

| **chromosome** | **start** | **stop** | **average ratio/CBS segment** | **oligos/segment** |
| --- | --- | --- | --- | --- |
| 1 | 564424 | 2765252 | -0,9372 | 314 |
| 1 | 2771708 | 45090613 | -0,0931 | 7033 |
| 1 | 45099320 | 45213061 | 0,4473 | 24 |
| 1 | 45220436 | 54757379 | -0,0804 | 1711 |
| 1 | 54761803 | 54820976 | -0,9445 | 12 |
| 1 | 54829146 | 54908093 | -0,0841 | 16 |
| 1 | 54925102 | 54968441 | 0,3568 | 9 |
| 1 | 54975155 | 60391772 | -0,0553 | 886 |
| 1 | 60397586 | 60490676 | 0,8977 | 19 |
| 1 | 60494065 | 72269817 | -0,0773 | 1905 |
| 1 | 72275234 | 72540780 | 0,5071 | 59 |
| 1 | 72546720 | 76519588 | -0,0161 | 488 |
| 1 | 76523719 | 79027466 | -0,0959 | 447 |
| 1 | 79031131 | 107586214 | -0,037 | 3662 |
| 1 | 107590003 | 121225278 | -0,0745 | 2157 |
| 1 | 121232209 | 121350930 | 0,0355 | 20 |
| 1 | 142617943 | 149063254 | -0,3663 | 314 |
| 1 | 149079747 | 149821717 | -0,1461 | 25 |
| 1 | 149821810 | 152553314 | 0,0374 | 568 |
| 1 | 152556449 | 152581944 | -13.107 | 6 |
| 1 | 152586234 | 153014245 | 0,0355 | 90 |
| 1 | 153017870 | 153171653 | 0,2006 | 26 |
| 1 | 153177158 | 158533302 | 0,0167 | 1065 |
| 1 | 158538546 | 158558512 | 0,4145 | 6 |
| 1 | 158564876 | 160778746 | 0,0515 | 407 |
| 1 | 160785763 | 161253884 | -0,0355 | 97 |
| 1 | 161258236 | 165313207 | 0,0488 | 637 |
| 1 | 165319559 | 166140002 | -0,0419 | 159 |
| 1 | 166142017 | 169216702 | 0,0297 | 540 |
| 1 | 169227144 | 169254178 | 0,4513 | 6 |
| 1 | 169256516 | 171162610 | 0,0628 | 309 |
| 1 | 171166163 | 183278859 | -0,0095 | 2056 |
| 1 | 183286134 | 183384583 | 0,6278 | 21 |
| 1 | 183392116 | 199988176 | 0,0173 | 1963 |
| 1 | 199992476 | 203095156 | -0,0453 | 589 |
| 1 | 203098367 | 206913210 | -0,106 | 636 |
| 1 | 206919643 | 207423016 | -0,0528 | 100 |
| 1 | 207438995 | 226632012 | -0,1293 | 2863 |
| 1 | 226636207 | 231439350 | -0,186 | 784 |
| 1 | 231449141 | 242357267 | -0,1329 | 1761 |
| 1 | 242364270 | 242423027 | 0,2927 | 12 |
| 1 | 242428075 | 243283519 | -0,1576 | 84 |
| 1 | 243288228 | 243327119 | 0,1849 | 9 |
| 1 | 243330530 | 248043631 | -0,1338 | 890 |
| 1 | 248047438 | 249218792 | -0,0968 | 173 |
| 2 | 17019 | 24516594 | -0,0999 | 2994 |
| 2 | 24520261 | 24544287 | -0,8484 | 5 |
| 2 | 24548386 | 33513211 | -0,1257 | 1688 |
| 2 | 33516865 | 40840415 | -0,086 | 1407 |
| 2 | 40845287 | 41358913 | 0,0198 | 101 |
| 2 | 41365745 | 47167732 | -0,0967 | 940 |
| 2 | 47170612 | 50129960 | -0,127 | 443 |
| 2 | 50134899 | 53679621 | -0,0118 | 496 |
| 2 | 53688330 | 77228947 | -0,0979 | 3510 |
| 2 | 77233582 | 84650871 | -0,0488 | 808 |
| 2 | 84656556 | 117979304 | -0,1049 | 3506 |
| 2 | 118005754 | 118111203 | 0,3882 | 6 |
| 2 | 118137847 | 118207105 | -0,194 | 7 |
| 2 | 118249154 | 118398138 | 0,3571 | 13 |
| 2 | 118422232 | 119694513 | -0,0234 | 121 |
| 2 | 119700566 | 137101823 | -0,1209 | 2120 |
| 2 | 137117179 | 138376783 | -0,0451 | 195 |
| 2 | 138385809 | 139513968 | -0,1445 | 136 |
| 2 | 139517753 | 144743633 | -0,0573 | 739 |
| 2 | 144751679 | 144838455 | -0,9092 | 17 |
| 2 | 144844175 | 148360482 | -0,0041 | 319 |
| 2 | 148372337 | 150038389 | -0,1051 | 249 |
| 2 | 150044993 | 169892860 | -0,077 | 2740 |
| 2 | 169897111 | 169918656 | 0,4028 | 4 |
| 2 | 169923077 | 170139541 | -0,0228 | 47 |
| 2 | 170142954 | 180088340 | -0,1103 | 1660 |
| 2 | 180093318 | 200018455 | -0,0698 | 2491 |
| 2 | 200031456 | 200040131 | 0,705 | 2 |
| 2 | 200052050 | 201638432 | -0,0599 | 232 |
| 2 | 201642929 | 210790660 | -0,1093 | 1542 |
| 2 | 210795226 | 215691790 | -0,0505 | 786 |
| 2 | 215696001 | 220541184 | -0,1414 | 820 |
| 2 | 220545425 | 231731954 | -0,087 | 1470 |
| 2 | 231738225 | 243041364 | -0,1382 | 1854 |
| 3 | 62075 | 611695 | 0,5881 | 89 |
| 3 | 617415 | 660813 | -0,0445 | 6 |
| 3 | 666257 | 2986409 | 0,576 | 382 |
| 3 | 2993515 | 6364740 | 0,4962 | 447 |
| 3 | 6375270 | 8460961 | 0,5795 | 290 |
| 3 | 8465947 | 9048409 | -0,4154 | 92 |
| 3 | 9057345 | 17309188 | 0,4302 | 1432 |
| 3 | 17316656 | 31720112 | 0,5249 | 1788 |
| 3 | 31725033 | 33017383 | 0,4049 | 228 |
| 3 | 33025609 | 36390055 | 0,515 | 368 |
| 3 | 36403910 | 48949514 | 0,4265 | 2130 |
| 3 | 48953064 | 50751321 | 0,3611 | 388 |
| 3 | 50754233 | 51259100 | 0,4923 | 81 |
| 3 | 51263154 | 52568641 | 0,3516 | 268 |
| 3 | 52571548 | 54499644 | 0,4346 | 346 |
| 3 | 54501177 | 60360280 | 0,4936 | 976 |
| 3 | 60367507 | 60416652 | -0,4059 | 11 |
| 3 | 60422896 | 60544760 | -35.902 | 25 |
| 3 | 60551364 | 60637427 | -0,3271 | 19 |
| 3 | 60642727 | 75379220 | 0,4797 | 2123 |
| 3 | 75386916 | 75998947 | 0,104 | 23 |
| 3 | 76005691 | 86071641 | 0,546 | 1203 |
| 3 | 86075705 | 90309067 | 0,4935 | 421 |
| 3 | 93538467 | 106901887 | 0,1162 | 1589 |
| 3 | 106912804 | 107368692 | -0,4602 | 69 |
| 3 | 107372962 | 118890383 | 0,1025 | 1613 |
| 3 | 118894174 | 141713999 | 0,0602 | 3850 |
| 3 | 141724273 | 159089811 | 0,1176 | 2304 |
| 3 | 159094772 | 159286417 | -0,3235 | 39 |
| 3 | 159295188 | 162170817 | 0,0842 | 403 |
| 3 | 162181138 | 162504822 | 0,2124 | 21 |
| 3 | 162514534 | 162619141 | 0,6445 | 14 |
| 3 | 162630179 | 168856129 | 0,1784 | 668 |
| 3 | 168863349 | 169371879 | -0,4152 | 104 |
| 3 | 169375537 | 179985160 | 0,1153 | 1600 |
| 3 | 179993283 | 180511081 | 0,4588 | 68 |
| 3 | 180521519 | 189588477 | 0,0881 | 1416 |
| 3 | 189592668 | 189684755 | 10.389 | 18 |
| 3 | 189688623 | 194391959 | 0,1302 | 687 |
| 3 | 194396656 | 197845254 | 0,0804 | 647 |
| 4 | 45882 | 68211 | -0,3412 | 5 |
| 4 | 71552 | 582765 | 0,3559 | 93 |
| 4 | 595962 | 1988875 | 0,2661 | 239 |
| 4 | 1990411 | 3535755 | 0,3466 | 286 |
| 4 | 3574456 | 3631805 | 0,0006 | 15 |
| 4 | 3636834 | 8908708 | 0,3403 | 911 |
| 4 | 8910217 | 9766737 | -0,0695 | 13 |
| 4 | 9803496 | 10469317 | 0,4193 | 94 |
| 4 | 10473527 | 10492249 | -0,011 | 5 |
| 4 | 10497720 | 34787187 | 0,4607 | 2547 |
| 4 | 34803080 | 34814779 | -1.202 | 2 |
| 4 | 34833500 | 37698551 | 0,561 | 307 |
| 4 | 37701637 | 42572849 | 0,432 | 835 |
| 4 | 42577586 | 46135805 | 0,5309 | 346 |
| 4 | 46141199 | 58370271 | 0,4576 | 1374 |
| 4 | 58375445 | 69406027 | 0,5262 | 952 |
| 4 | 69414741 | 69483277 | -0,3134 | 8 |
| 4 | 69608683 | 78636124 | 0,5 | 1326 |
| 4 | 78639061 | 80402284 | 10.272 | 288 |
| 4 | 80418371 | 87247773 | 0,499 | 1015 |
| 4 | 87253172 | 87514551 | -0,4271 | 44 |
| 4 | 87519718 | 89660410 | 0,4699 | 394 |
| 4 | 89663348 | 91407236 | 0,5528 | 244 |
| 4 | 91415897 | 91779504 | 0,4819 | 70 |
| 4 | 91783492 | 91868931 | -0,4329 | 10 |
| 4 | 91876559 | 103867409 | 0,5194 | 1691 |
| 4 | 103880669 | 103988696 | 10.329 | 22 |
| 4 | 103994544 | 122740669 | 0,5008 | 2382 |
| 4 | 122741857 | 122741916 | -0,1282 | 5 |
| 4 | 122746386 | 124428903 | 0,4745 | 272 |
| 4 | 124437690 | 138640512 | 0,5259 | 1148 |
| 4 | 138663344 | 155779765 | 0,4771 | 2450 |
| 4 | 155788216 | 182641017 | 0,5338 | 3160 |
| 4 | 182657533 | 187591768 | 0,4695 | 841 |
| 4 | 187595209 | 190480866 | 0,5547 | 382 |
| 4 | 190488733 | 190678708 | -0,1249 | 25 |
| 4 | 190687570 | 190916678 | 0,4173 | 23 |
| 5 | 26142 | 144408 | 0,7817 | 15 |
| 5 | 147640 | 224141 | 10.284 | 14 |
| 5 | 226358 | 1879462 | 0,3158 | 247 |
| 5 | 1884742 | 7634436 | 0,4409 | 718 |
| 5 | 7639686 | 8582426 | -0,1336 | 112 |
| 5 | 8597424 | 17345514 | 0,4457 | 1213 |
| 5 | 17349181 | 31208919 | 0,5043 | 1227 |
| 5 | 31212141 | 46115086 | 0,4231 | 2089 |
| 5 | 49559920 | 59481493 | -0,089 | 1313 |
| 5 | 59493142 | 59799206 | 0,4531 | 42 |
| 5 | 59806227 | 80817739 | -0,0987 | 2802 |
| 5 | 80822032 | 121166942 | -0,0723 | 4621 |
| 5 | 121172629 | 121815022 | 0,039 | 93 |
| 5 | 121824665 | 125634960 | -0,0695 | 388 |
| 5 | 125650832 | 128628683 | -0,1077 | 448 |
| 5 | 128645470 | 131297998 | -0,0362 | 347 |
| 5 | 131302175 | 141076783 | -0,1197 | 1738 |
| 5 | 141082661 | 178706458 | 0,422 | 5372 |
| 5 | 178711746 | 180712263 | -0,1479 | 349 |
| 6 | 154130 | 279726 | -0,0649 | 20 |
| 6 | 283968 | 378956 | -0,2647 | 21 |
| 6 | 384100 | 3933095 | -0,0444 | 607 |
| 6 | 3939209 | 4008723 | 0,7584 | 17 |
| 6 | 4018128 | 20919550 | -0,0364 | 2449 |
| 6 | 20922535 | 21040431 | -0,9842 | 25 |
| 6 | 21043768 | 22469308 | -0,0939 | 172 |
| 6 | 22492351 | 29447624 | -0,034 | 1139 |
| 6 | 29451208 | 32450744 | -0,0717 | 591 |
| 6 | 32455274 | 32459543 | 0,732 | 2 |
| 6 | 32479770 | 44780763 | -0,069 | 2219 |
| 6 | 44789925 | 57380592 | -0,0308 | 1939 |
| 6 | 57384952 | 69644919 | 0,0182 | 891 |
| 6 | 69651252 | 90577465 | -0,0356 | 2860 |
| 6 | 90583578 | 96722233 | 0,0358 | 570 |
| 6 | 96727359 | 103720471 | -0,0345 | 848 |
| 6 | 103738390 | 103753939 | 0,9825 | 2 |
| 6 | 103766891 | 104391154 | 0,0074 | 45 |
| 6 | 104404390 | 112343408 | -0,0679 | 1264 |
| 6 | 112355152 | 143387979 | 0,5005 | 4355 |
| 6 | 143390477 | 161956316 | 0,4388 | 2845 |
| 6 | 161961388 | 166033526 | 0,5198 | 599 |
| 6 | 166042572 | 170911240 | 0,4472 | 747 |
| 7 | 44944 | 7044369 | 0,082 | 1114 |
| 7 | 7058784 | 17635690 | -0,1969 | 1456 |
| 7 | 17651263 | 24252337 | -0,2561 | 960 |
| 7 | 24268584 | 24289016 | -0,9348 | 4 |
| 7 | 24292897 | 56169989 | -0,2669 | 4545 |
| 7 | 56174829 | 57621419 | -0,1841 | 95 |
| 7 | 62452971 | 66204857 | 0,3436 | 376 |
| 7 | 66213176 | 72256476 | 0,3972 | 836 |
| 7 | 72274004 | 75956504 | 0,3046 | 430 |
| 7 | 75963558 | 76004543 | 0,6053 | 8 |
| 7 | 76009765 | 76493213 | 0,2973 | 27 |
| 7 | 76511777 | 78932019 | 0,4677 | 415 |
| 7 | 78936886 | 83252795 | 0,5193 | 597 |
| 7 | 83259863 | 97455574 | 0,4521 | 2008 |
| 7 | 97462439 | 98495049 | 0,3201 | 148 |
| 7 | 98498161 | 99518748 | 0,3999 | 207 |
| 7 | 99526250 | 102335349 | 0,3261 | 511 |
| 7 | 102339318 | 102713643 | 0,496 | 64 |
| 7 | 102718259 | 103030942 | 0,382 | 40 |
| 7 | 103038450 | 103108416 | -0,0575 | 12 |
| 7 | 103113306 | 111100798 | 0,4383 | 1548 |
| 7 | 111105151 | 112440163 | 0,3717 | 261 |
| 7 | 112445944 | 115568584 | 0,4777 | 601 |
| 7 | 115576479 | 115628125 | 0,8998 | 11 |
| 7 | 115644711 | 117881952 | 0,4153 | 454 |
| 7 | 117885689 | 127086031 | 0,4797 | 1729 |
| 7 | 127103216 | 133739575 | 0,3679 | 1324 |
| 7 | 133743247 | 137584783 | 0,4396 | 762 |
| 7 | 137588161 | 144106867 | 0,3863 | 1128 |
| 7 | 144113548 | 147676223 | 0,4447 | 703 |
| 7 | 147680449 | 159118566 | 0,3814 | 1963 |
| 8 | 161472 | 30748085 | -0,218 | 4445 |
| 8 | 30775975 | 36642937 | 0,3559 | 740 |
| 8 | 36649900 | 39040659 | 0,2701 | 355 |
| 8 | 39041618 | 41362439 | 0,3764 | 310 |
| 8 | 41367054 | 41662292 | 0,2376 | 70 |
| 8 | 41669405 | 42378936 | 0,3396 | 130 |
| 8 | 42385169 | 48947064 | 0,2535 | 337 |
| 8 | 48950308 | 54758375 | 0,3475 | 712 |
| 8 | 54764541 | 56097345 | 0,287 | 173 |
| 8 | 56101010 | 56147142 | 0,8521 | 10 |
| 8 | 56150183 | 75595762 | 0,3097 | 2638 |
| 8 | 75601849 | 79902777 | 0,3821 | 406 |
| 8 | 79934854 | 82189022 | 0,2838 | 286 |
| 8 | 82193925 | 89021920 | 0,3963 | 798 |
| 8 | 89034245 | 111945939 | 0,3141 | 3226 |
| 8 | 111951212 | 117450545 | 0,3824 | 559 |
| 8 | 117469787 | 131184215 | 0,3159 | 1916 |
| 8 | 131188702 | 131396631 | -0,2572 | 43 |
| 8 | 131399965 | 132070900 | 0,2908 | 92 |
| 8 | 132080967 | 133294492 | 0,3838 | 129 |
| 8 | 133304003 | 142202491 | 0,2964 | 1165 |
| 8 | 142205751 | 142507507 | 0,22 | 61 |
| 8 | 142516305 | 142531643 | -0,3835 | 4 |
| 8 | 142539571 | 146057499 | 0,1874 | 587 |
| 8 | 146060914 | 146294098 | 0,272 | 37 |
| 9 | 204193 | 254712 | -0,3802 | 10 |
| 9 | 258399 | 1075540 | 0,5076 | 157 |
| 9 | 1079463 | 3512929 | 0,5746 | 351 |
| 9 | 3519377 | 6965025 | 0,521 | 593 |
| 9 | 6969619 | 13454718 | 0,5824 | 829 |
| 9 | 13462689 | 33023850 | 0,4988 | 2530 |
| 9 | 33028610 | 44259464 | 0,4171 | 993 |
| 9 | 45469331 | 72882902 | -0,0992 | 340 |
| 9 | 72888048 | 90453069 | -0,0572 | 2460 |
| 9 | 90463564 | 103776453 | -0,0984 | 2020 |
| 9 | 103781780 | 108275138 | -0,0053 | 560 |
| 9 | 108282767 | 109093379 | -0,0714 | 103 |
| 9 | 109102398 | 109538143 | -0,4361 | 50 |
| 9 | 109564685 | 116059425 | -0,0702 | 1062 |
| 9 | 116060890 | 118719495 | -0,1279 | 432 |
| 9 | 118733325 | 119419531 | -0,0702 | 109 |
| 9 | 119422945 | 119478493 | -0,819 | 12 |
| 9 | 119484432 | 123222841 | -0,06 | 409 |
| 9 | 123227567 | 123239453 | 0,3167 | 3 |
| 9 | 123246349 | 123776165 | -0,0665 | 105 |
| 9 | 123782336 | 136194644 | -0,1153 | 2306 |
| 9 | 136199836 | 140999928 | -0,1593 | 879 |
| 9 | 141008863 | 141025921 | 0,128 | 3 |
| 10 | 136361 | 75915820 | -0,3607 | 10179 |
| 10 | 75923421 | 76017728 | -12.043 | 17 |
| 10 | 76022993 | 82876632 | -0,3957 | 1089 |
| 10 | 82883786 | 82891283 | -4.461 | 2 |
| 10 | 82897174 | 135434178 | -0,372 | 8018 |
| 11 | 200215 | 945857 | -0,1084 | 147 |
| 11 | 952319 | 1120371 | -0,2433 | 42 |
| 11 | 1122082 | 1689081 | -0,0805 | 99 |
| 11 | 1696419 | 1700295 | 0,641 | 2 |
| 11 | 1704417 | 3247952 | -0,0975 | 293 |
| 11 | 3254177 | 4698065 | -0,0116 | 177 |
| 11 | 4701501 | 5375725 | 0,0757 | 142 |
| 11 | 5385301 | 6278388 | 0,0061 | 191 |
| 11 | 6282812 | 6676031 | -0,111 | 75 |
| 11 | 6683603 | 15887559 | -0,0004 | 1526 |
| 11 | 15903944 | 16757938 | 0,0813 | 139 |
| 11 | 16761426 | 21930782 | -0,0011 | 909 |
| 11 | 21955890 | 31696685 | 0,0647 | 1147 |
| 11 | 31705076 | 36472190 | 0,0008 | 792 |
| 11 | 36479186 | 42861910 | 0,059 | 540 |
| 11 | 42891032 | 48328800 | -0,0352 | 871 |
| 11 | 48333517 | 56979925 | 0,0618 | 487 |
| 11 | 56984320 | 57844092 | -0,0598 | 163 |
| 11 | 57846216 | 60638286 | 0,0333 | 484 |
| 11 | 60641691 | 66704236 | -0,0565 | 1197 |
| 11 | 66712836 | 66784073 | 0,3157 | 14 |
| 11 | 66789054 | 75270440 | -0,0393 | 1388 |
| 11 | 75276723 | 88503772 | 0,0124 | 1930 |
| 11 | 88503862 | 92111218 | 0,0892 | 376 |
| 11 | 92115136 | 96579366 | 0,0137 | 705 |
| 11 | 96589279 | 100078761 | 0,075 | 378 |
| 11 | 100083897 | 104654343 | 0,0268 | 633 |
| 11 | 104666540 | 104723863 | -0,1244 | 5 |
| 11 | 104737503 | 134945165 | -0,5507 | 4684 |
| 12 | 87496 | 163643 | -0,119 | 2 |
| 12 | 189561 | 3058678 | 0,275 | 557 |
| 12 | 3062397 | 3326298 | 0,1614 | 52 |
| 12 | 3330358 | 3821354 | 0,2586 | 83 |
| 12 | 3828827 | 6137493 | 0,3242 | 375 |
| 12 | 6143121 | 9246041 | 0,268 | 516 |
| 12 | 9251676 | 20579905 | 0,3424 | 1653 |
| 12 | 20588366 | 24348339 | 0,4112 | 599 |
| 12 | 24349919 | 26595737 | 0,3451 | 369 |
| 12 | 26601308 | 26658253 | -0,0804 | 13 |
| 12 | 26661401 | 31104993 | 0,347 | 603 |
| 12 | 31109037 | 38598586 | 0,2965 | 505 |
| 12 | 38614223 | 39226750 | 0,5545 | 74 |
| 12 | 39230208 | 39242397 | 0,6758 | 4 |
| 12 | 39247950 | 42123197 | 0,9296 | 435 |
| 12 | 42142001 | 45747247 | 0,8597 | 508 |
| 12 | 45752353 | 71209840 | -0,0964 | 4091 |
| 12 | 71216003 | 96230394 | -0,0547 | 3112 |
| 12 | 96238905 | 98959431 | -0,0829 | 371 |
| 12 | 98964569 | 99000591 | -11.097 | 10 |
| 12 | 99007493 | 99163933 | -0,1467 | 32 |
| 12 | 99166844 | 102028407 | -0,0701 | 523 |
| 12 | 102035013 | 126017452 | -0,1283 | 3851 |
| 12 | 126021808 | 131652868 | -0,0539 | 747 |
| 12 | 131675403 | 133779076 | -0,161 | 360 |
| 13 | 19296544 | 23664269 | -0,3906 | 645 |
| 13 | 23681101 | 32301631 | 0,0928 | 1476 |
| 13 | 32307644 | 38917123 | 0,1348 | 1082 |
| 13 | 38924694 | 41410023 | 0,0778 | 427 |
| 13 | 41426267 | 45602355 | 0,1285 | 733 |
| 13 | 45606448 | 51060533 | 0,081 | 956 |
| 13 | 51070214 | 51269033 | 0,1253 | 30 |
| 13 | 51273130 | 51349850 | 0,5595 | 27 |
| 13 | 51354340 | 51423054 | 0,1682 | 15 |
| 13 | 51429791 | 51483678 | 0,4809 | 7 |
| 13 | 51486815 | 53742120 | 0,0953 | 407 |
| 13 | 53755412 | 75713911 | 0,1726 | 2282 |
| 13 | 75718137 | 81396797 | 0,0905 | 788 |
| 13 | 81417735 | 94867826 | 0,1642 | 1469 |
| 13 | 94871308 | 98595580 | 0,0782 | 695 |
| 13 | 98599596 | 104075929 | 0,0415 | 1066 |
| 13 | 104083670 | 109249951 | 0,1196 | 567 |
| 13 | 109257434 | 113219512 | 0,051 | 631 |
| 13 | 113223769 | 115107245 | -0,0126 | 350 |
| 14 | 19376762 | 20502940 | -0,3957 | 43 |
| 14 | 20512842 | 20571401 | 0,149 | 10 |
| 14 | 20582059 | 20745095 | -0,4159 | 32 |
| 14 | 20754595 | 20802272 | 0,0792 | 19 |
| 14 | 20805115 | 20875310 | -0,4946 | 20 |
| 14 | 20880746 | 23229612 | 0,1232 | 502 |
| 14 | 23237589 | 24912364 | 0,0653 | 342 |
| 14 | 24925385 | 30361288 | 0,1692 | 583 |
| 14 | 30366077 | 32830929 | 0,1174 | 402 |
| 14 | 32836845 | 33792739 | 0,1637 | 195 |
| 14 | 33797063 | 39780117 | 0,0993 | 997 |
| 14 | 39783134 | 41787504 | 0,1393 | 149 |
| 14 | 41793382 | 42068723 | 0,3834 | 24 |
| 14 | 42075453 | 49958953 | 0,1421 | 793 |
| 14 | 49973776 | 50061016 | 0,4646 | 14 |
| 14 | 50064260 | 50176571 | 0,134 | 24 |
| 14 | 50183001 | 54875298 | 0,0959 | 747 |
| 14 | 54875477 | 60047954 | 0,053 | 889 |
| 14 | 60052486 | 64430700 | 0,0847 | 739 |
| 14 | 64434440 | 64470034 | 0,7116 | 9 |
| 14 | 64473934 | 67323167 | 0,4555 | 490 |
| 14 | 67333561 | 67449489 | 0,0679 | 23 |
| 14 | 67453057 | 68405559 | 0,4438 | 209 |
| 14 | 68409163 | 68981584 | -0,4565 | 122 |
| 14 | 68985568 | 78769514 | 0,4182 | 1892 |
| 14 | 78775394 | 90381459 | 0,4842 | 1494 |
| 14 | 90386270 | 93609142 | 0,393 | 646 |
| 14 | 93618178 | 100568816 | 0,4424 | 1033 |
| 14 | 100571169 | 104992852 | 0,3872 | 909 |
| 14 | 104996963 | 105133748 | 0,663 | 28 |
| 14 | 105142894 | 106526266 | 0,3163 | 232 |
| 14 | 106531557 | 106768453 | 0,8755 | 31 |
| 14 | 106772041 | 107287505 | 0,4719 | 98 |
| 15 | 20102541 | 22756709 | -0,166 | 88 |
| 15 | 22765628 | 35459991 | -0,4556 | 1771 |
| 15 | 35465993 | 40072127 | -0,7199 | 592 |
| 15 | 40075470 | 40625153 | -0,7998 | 123 |
| 15 | 40628386 | 40667333 | -11.212 | 12 |
| 15 | 40671929 | 50389968 | -0,7322 | 1859 |
| 15 | 50399128 | 50427567 | -17.428 | 8 |
| 15 | 50431866 | 74182845 | -0,7521 | 4359 |
| 15 | 74187845 | 74305036 | -11.911 | 27 |
| 15 | 74308963 | 102394710 | -0,7425 | 4675 |
| 15 | 102399760 | 102480888 | -0,196 | 5 |
| 16 | 93628 | 628939 | 0,056 | 108 |
| 16 | 633597 | 1412865 | -0,0445 | 149 |
| 16 | 1421108 | 2207456 | 0,0563 | 160 |
| 16 | 2210351 | 2241182 | -0,2577 | 7 |
| 16 | 2248552 | 2841269 | 0,0295 | 106 |
| 16 | 2849908 | 3641123 | 0,088 | 152 |
| 16 | 3649887 | 3672598 | 0,3954 | 5 |
| 16 | 3674286 | 4021542 | 0,0951 | 66 |
| 16 | 4026581 | 5301942 | 0,0403 | 225 |
| 16 | 5348722 | 11098095 | 0,1104 | 849 |
| 16 | 11102191 | 11231125 | -0,4045 | 28 |
| 16 | 11237852 | 31955135 | 0,0881 | 2798 |
| 16 | 32471625 | 59056504 | 0,0065 | 1951 |
| 16 | 59066428 | 66098014 | 0,0736 | 624 |
| 16 | 66117416 | 69238987 | -0,0286 | 613 |
| 16 | 69245922 | 69304080 | -0,3889 | 15 |
| 16 | 69308738 | 69942298 | -0,0075 | 133 |
| 16 | 69944984 | 69976962 | -0,1977 | 15 |
| 16 | 70048808 | 70125181 | 0,0782 | 8 |
| 16 | 70128195 | 70193942 | -0,3222 | 8 |
| 16 | 70280883 | 70478460 | -0,0742 | 43 |
| 16 | 70483059 | 70834000 | -0,5033 | 72 |
| 16 | 70839601 | 71167245 | -0,1909 | 14 |
| 16 | 71205672 | 73549652 | -0,5019 | 352 |
| 16 | 73554745 | 77912848 | -0,4384 | 593 |
| 16 | 77918547 | 78437672 | -0,5038 | 104 |
| 16 | 78445571 | 78459295 | -1.414 | 4 |
| 16 | 78466362 | 78620872 | -45.187 | 27 |
| 16 | 78625861 | 79009057 | -14.332 | 72 |
| 16 | 79015710 | 82920558 | -0,4624 | 530 |
| 16 | 82926663 | 83463890 | -14.239 | 108 |
| 16 | 83469809 | 87889547 | -0,478 | 748 |
| 16 | 87904877 | 87971998 | 0,1416 | 8 |
| 16 | 87977509 | 88710656 | -0,0204 | 89 |
| 16 | 88714940 | 88778340 | -0,3459 | 15 |
| 16 | 88785985 | 89369211 | -0,0261 | 107 |
| 16 | 89376186 | 89398694 | -0,4455 | 6 |
| 16 | 89403135 | 90163114 | 0,0261 | 156 |
| 17 | 47546 | 1959657 | 0,0582 | 381 |
| 17 | 1963124 | 26113910 | -0,4455 | 3357 |
| 17 | 26120287 | 30135129 | -0,398 | 753 |
| 17 | 30137075 | 30142017 | -0,0063 | 3 |
| 17 | 30143833 | 30262956 | -0,3845 | 26 |
| 17 | 30289911 | 32385091 | -0,269 | 402 |
| 17 | 32394112 | 32858733 | -0,428 | 58 |
| 17 | 32876171 | 32954915 | 0,0325 | 15 |
| 17 | 32958056 | 36573051 | -0,4335 | 524 |
| 17 | 36578268 | 40881014 | -0,5462 | 846 |
| 17 | 40887394 | 40904742 | -2.774 | 5 |
| 17 | 40910621 | 81108062 | -0,1361 | 6401 |
| 18 | 14316 | 14978134 | -0,1907 | 2213 |
| 18 | 15072735 | 31659244 | 0,2007 | 1797 |
| 18 | 31666325 | 31730206 | 0,6455 | 13 |
| 18 | 31733233 | 33507637 | 0,2183 | 258 |
| 18 | 33515212 | 40396404 | 0,1726 | 727 |
| 18 | 40402263 | 42437315 | 0,2167 | 200 |
| 18 | 42441709 | 43286858 | 0,8545 | 126 |
| 18 | 43293620 | 43435785 | 0,5377 | 38 |
| 18 | 43441088 | 43818386 | 0,1412 | 70 |
| 18 | 43823120 | 44447514 | 0,5361 | 107 |
| 18 | 44457858 | 45423225 | 0,6139 | 138 |
| 18 | 45428356 | 45933313 | 0,5251 | 69 |
| 18 | 45953441 | 46074744 | 10.519 | 14 |
| 18 | 46079917 | 47753653 | 0,5452 | 298 |
| 18 | 47763851 | 47802768 | 0,3245 | 8 |
| 18 | 47810513 | 48069652 | -0,2889 | 31 |
| 18 | 48069593 | 50332445 | -0,3709 | 305 |
| 18 | 50340841 | 51240146 | -0,2769 | 150 |
| 18 | 51259987 | 51486368 | -11.899 | 11 |
| 18 | 51516889 | 54134880 | -0,3241 | 308 |
| 18 | 54150336 | 54214917 | 0,2907 | 3 |
| 18 | 54235316 | 62079166 | -0,3504 | 1185 |
| 18 | 62088545 | 70866904 | -0,2832 | 822 |
| 18 | 70874102 | 72351388 | -0,3617 | 162 |
| 18 | 72357349 | 72721380 | -1.267 | 74 |
| 18 | 72726415 | 78010032 | -0,355 | 764 |
| 19 | 259395 | 19699438 | -0,1159 | 3886 |
| 19 | 19703497 | 19748113 | 0,1674 | 9 |
| 19 | 19751495 | 21746048 | 0,4107 | 291 |
| 19 | 21749989 | 24366074 | -0,08 | 320 |
| 19 | 27853207 | 28419244 | 0,2798 | 16 |
| 19 | 28431784 | 28713443 | -0,9344 | 18 |
| 19 | 28727825 | 29993707 | -0,4937 | 151 |
| 19 | 29998724 | 31433571 | -0,1696 | 179 |
| 19 | 31448423 | 45255236 | 0,339 | 2300 |
| 19 | 45258255 | 51454441 | 0,278 | 1251 |
| 19 | 51459904 | 54402720 | 0,3428 | 631 |
| 19 | 54405781 | 56384205 | 0,6683 | 369 |
| 19 | 56388347 | 56388401 | 0,035 | 5 |
| 19 | 56391396 | 57211442 | 0,7146 | 163 |
| 19 | 57221883 | 57669059 | 12.747 | 76 |
| 19 | 57671961 | 57775778 | -0,2097 | 22 |
| 19 | 57780334 | 58528678 | 0,3069 | 158 |
| 19 | 58534319 | 58595237 | 0,7244 | 15 |
| 19 | 58599307 | 59047185 | 0,239 | 97 |
| 19 | 59057042 | 59095418 | 0,105 | 10 |
| 20 | 67778 | 32872383 | -0,313 | 4507 |
| 20 | 32876643 | 62949149 | 0,0785 | 4808 |
| 21 | 9832448 | 14420674 | -0,1702 | 37 |
| 21 | 14513884 | 18075132 | 0,0417 | 531 |
| 21 | 18081105 | 27066463 | 0,1073 | 1138 |
| 21 | 27071174 | 27943451 | -0,0068 | 169 |
| 21 | 27946129 | 30452517 | 0,0769 | 283 |
| 21 | 30457451 | 38512877 | 0,0044 | 1650 |
| 21 | 38517340 | 38520847 | -0,8775 | 2 |
| 21 | 38525923 | 44236675 | 0,0188 | 1189 |
| 21 | 44237499 | 47977171 | -0,0453 | 806 |
| 21 | 47983692 | 48098603 | 0,0458 | 28 |
| 22 | 16133474 | 19699800 | 0,0199 | 489 |
| 22 | 19702774 | 22065700 | -0,0364 | 380 |
| 22 | 22069249 | 24337667 | 0,0212 | 473 |
| 22 | 24347959 | 24405300 | -0,3679 | 11 |
| 22 | 24406990 | 27860314 | 0,0057 | 796 |
| 22 | 27863589 | 29121120 | 0,0661 | 282 |
| 22 | 29127855 | 30572839 | 0,0118 | 325 |
| 22 | 30575213 | 47297609 | -0,1065 | 3621 |
| 22 | 47303158 | 47377859 | 0,4739 | 17 |
| 22 | 47381306 | 48910248 | -0,0966 | 255 |
| 22 | 48914944 | 49953340 | 0,4008 | 198 |
| 22 | 49958215 | 51224252 | -0,1279 | 270 |
| X | 61091 | 1558421 | -0,3917 | 391 |
| X | 1559743 | 1566850 | 0,6657 | 3 |
| X | 1569356 | 8590043 | -0,3912 | 1122 |
| X | 8593486 | 25323709 | -0,4323 | 2667 |
| X | 25337964 | 32172447 | -0,3893 | 981 |
| X | 32175347 | 34761265 | -0,2821 | 380 |
| X | 34776210 | 48923131 | -0,4177 | 1990 |
| X | 48928825 | 48942873 | -42.092 | 4 |
| X | 48949306 | 81613609 | -0,423 | 3289 |
| X | 81660268 | 84184306 | -0,3191 | 251 |
| X | 84189686 | 87292534 | -0,3983 | 416 |
| X | 87303161 | 87314924 | 0,7785 | 2 |
| X | 87318268 | 109353575 | -0,4031 | 2482 |
| X | 109358110 | 109515685 | -43.246 | 33 |
| X | 109522397 | 112776395 | -0,4798 | 510 |
| X | 112787783 | 117252737 | -0,3912 | 535 |
| X | 117259873 | 123867972 | -0,4536 | 991 |
| X | 123871038 | 136328213 | -0,3997 | 1755 |
| X | 136342979 | 148568845 | 0,5262 | 1351 |
| X | 148572165 | 152697477 | 0,4311 | 663 |
| X | 152702218 | 153254143 | 0,2796 | 118 |
| X | 153261198 | 155226048 | 0,4052 | 347 |
| X | 155228914 | 155257126 | -0,202 | 10 |
| Y | 2650450 | 59031480 | 0,0403 | 1274 |

**Supplementary Table 4: Coordinates of 75 HaCaT/SM specific chromosomal breakpoints at base resolution.** Genomic coordinates refer to hg19. Only breakpoints with at least three independent breakpoint spanning reads are shown.

| chr1 | 54759566 | |
| --- | --- | --- |
| chr1 | 54826162 | |
| chr1 | 54912991 | |
| chr1 | 183285937 | |
| chr1 | 247095055 | |
| chr1 | 247095088 | |
| chr10 | 65230347 | |
| chr10 | 65308444 | |
| chr10 | 75917561 | |
| chr10 | 91659782 | |
| chr10 | 91747319 | |
| chr11 | 104629351 | |
| chr11 | | 104637198 |
| chr12 | | 45747395 |
| chr12 | | 64448812 |
| chr12 | | 64556630 |
| chr12 | | 120037012 |
| chr12 | | 120051705 |
| chr14 | | 64436819 |
| chr14 | | 67326096 |
| chr14 | | 68406080 |
| chr14 | | 68984072 |
| chr14 | | 82961814 |
| chr14 | | 82989022 |
| chr15 | | 50392312 |
| chr15 | | 50431098 |
| chr15 | | 101156197 |
| chr15 | | 101366678 |
| chr16 | | 3643437 |
| chr16 | | 3683204 |
| chr16 | | 11098592 |
| chr16 | | 11234231 |
| chr16 | | 78384899 |
| chr16 | | 78465122 |
| chr18 | | 46076880 |
| chr18 | | 47805380 |
| chr18 | | 51253116 |
| chr18 | | 51506470 |
| chr18 | | 72354212 |
| chr18 | | 72722522 |
| chr2 | | 144744258 |
| chr2 | | 144744259 |
| chr2 | | 200025377 |
| chr2 | | 200042982 |
| chr20 | | 10454242 |
| chr20 | | 10500956 |
| chr20 | | 32869213 |
| chr20 | | 32872591 |
| chr22 | | 47298315 |
| chr22 | | 47381192 |
| chr22 | | 48913970 |
| chr22 | | 49956887 |
| chr3 | | 8464062 |
| chr3 | | 9050624 |
| chr3 | | 61931782 |
| chr3 | | 62067080 |
| chr4 | | 87251010 |
| chr4 | | 87515535 |
| chr4 | | 91780236 |
| chr4 | | 91869857 |
| chr4 | | 175244845 |
| chr4 | | 175437185 |
| chr5 | | 7638951 |
| chr5 | | 8596755 |
| chr5 | | 66638384 |
| chr5 | | 67995925 |
| chr7 | | 7020670 |
| chr7 | | 24268532 |
| chr7 | | 24289254 |
| chr7 | | 110723519 |
| chr7 | | 110826335 |
| chr8 | | 5968310 |
| chr8 | | 6016526 |
| chrX | | 109357467 |
| chrX | | 109521582 |

| **Supplementary Table 5**: Sequence Read Statistic | |  |
| --- | --- | --- |
|  |  |  |
|  | **HaCaT** | **HaCaT/SM** |
| Total Reads | 909525746 | 749250016 |
| Reads Mapped: Unique | 893765774 | 736919966 |
| Reads Mapped: Ambigous | 14257922 | 10627452 |
| Reads Unmapped | 1502050 | 1702598 |
| Both Mates Mapped | 453935446 (99.8%) | 373703906 (99.8%) |
| Bases Mapped | 54124902315 | 44555988964 |
| Mean Read Length | 121.2 | 121.2 |
| Mismatches | 0.4 | 0.4 |
| Ref Bases Covered | 2909234026 | 2906106218 |
| Read Coverage Mean | 37.7 | 31.1 |
