# Supplementary figures and images for: Genomic Adaption and Mutational Patterns in a HaCaT Subline Resistant to Alkylating Agents and Ionizing Radiation
Source: Int J Mol Sci. 2021 Jan 24;22(3):1146. doi: 10.3390/ijms22031146 (PMC7865644; doi:10.3390/ijms22031146)

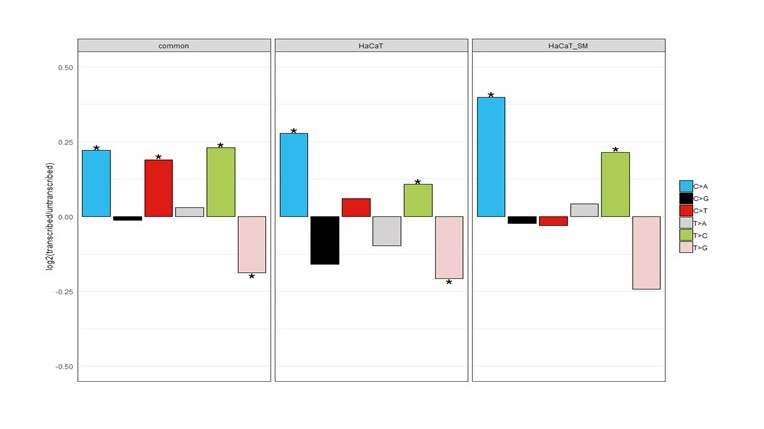

Supplement: Supplementary file 1 [file ijms-22-01146-s001.zip › SupplementaryFigure 4.jpg]

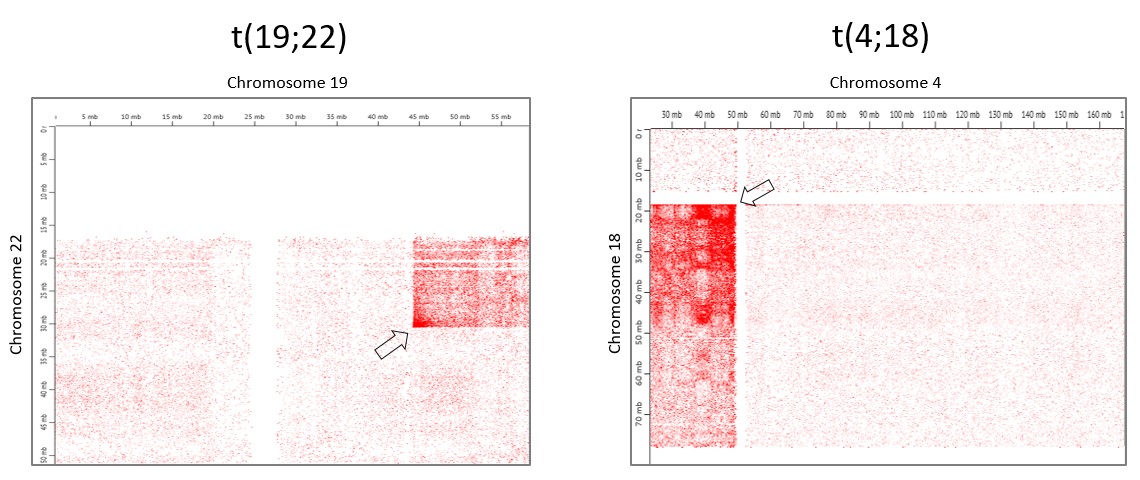

Supplement: Supplementary file 1 [file ijms-22-01146-s001.zip › SupplementaryFigure1.jpg]

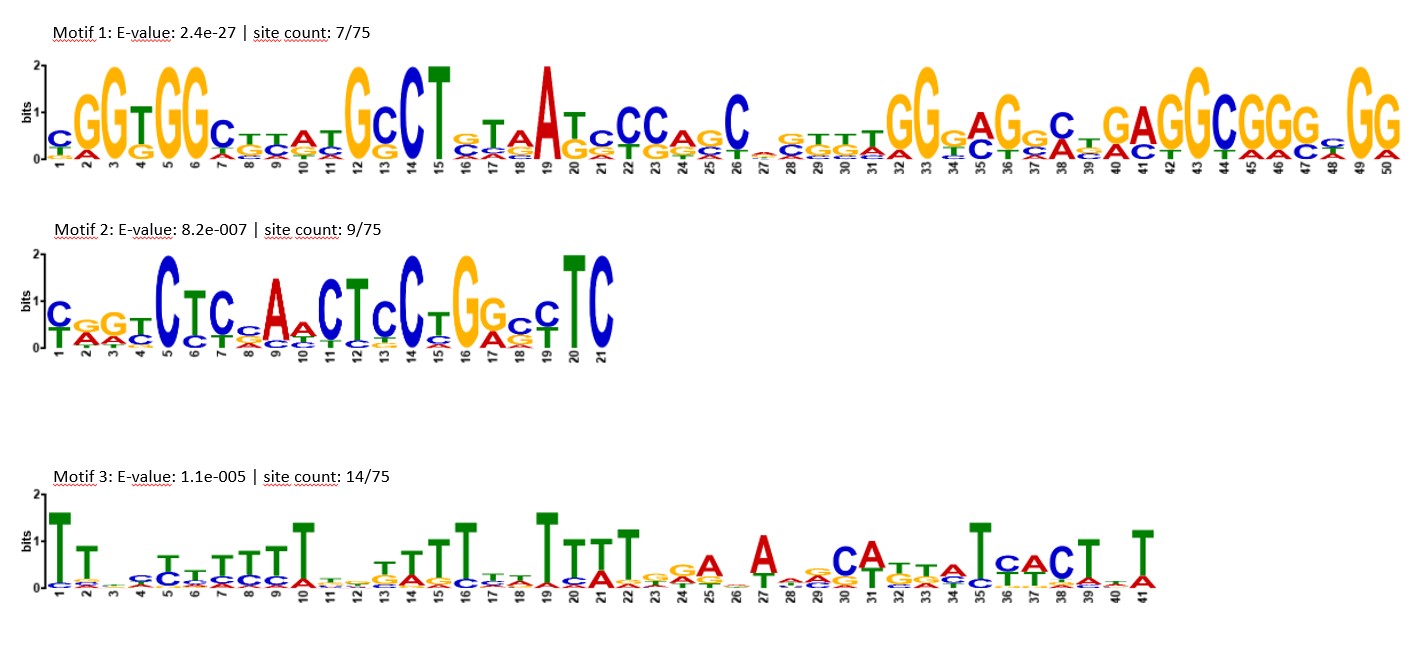

Supplement: Supplementary file 1 [file ijms-22-01146-s001.zip › SupplementaryFigure2.jpg]

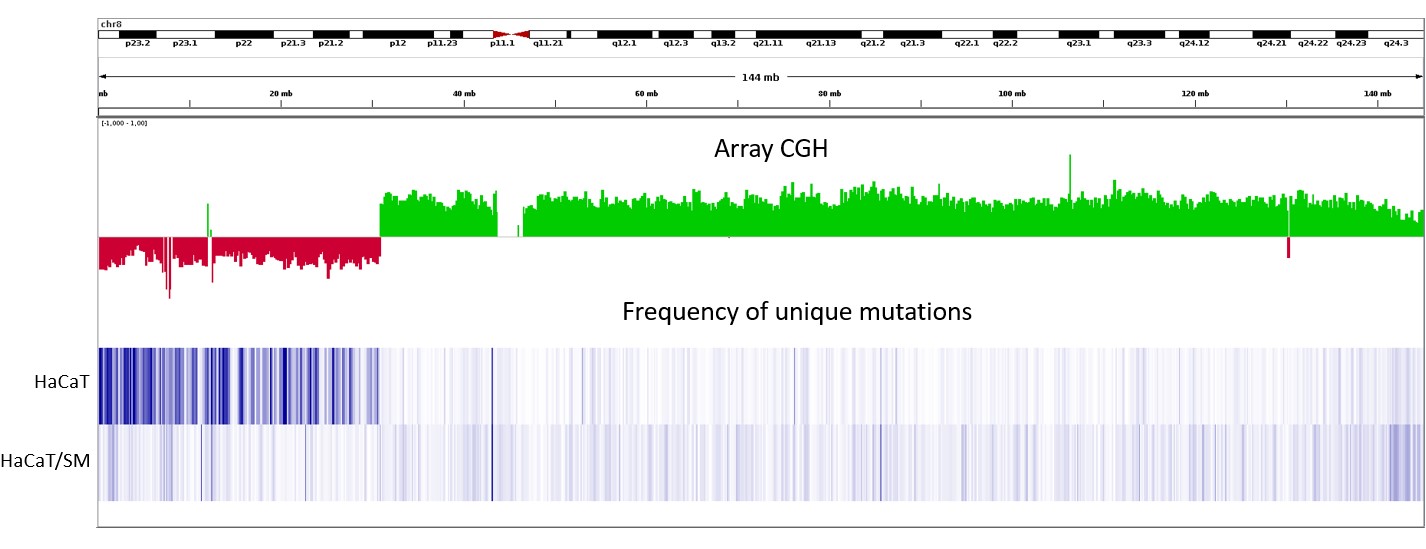

Supplement: Supplementary file 1 [file ijms-22-01146-s001.zip › SupplementaryFigure3.jpg]
